# Analysis and Screening of Commercialized Protein Supplements for Sports Practice

**DOI:** 10.3390/foods11213500

**Published:** 2022-11-03

**Authors:** Paloma Rodriguez-Lopez, Ascensión Rueda-Robles, Leticia Sánchez-Rodríguez, Rosa María Blanca-Herrera, Rosa María Quirantes-Piné, Isabel Borrás-Linares, Antonio Segura-Carretero, Jesús Lozano-Sánchez

**Affiliations:** 1 Department of Food Science and Nutrition, Campus Universitario s/n, University of Granada, 18071 Granada, Spain; 2 Research and Development Functional Food Centre (CIDAF), Health Science Technological Park, Edificio BioRegión, Avenida del Conocimiento 37, 18016 Granada, Spain; 3 Department of Analytical Chemistry, Faculty of Sciences, University of Granada, 18071 Granada, Spain

**Keywords:** protein supplements, analysis, digestibility, contaminants, food legislation, sports nutrition, nutrition label

## Abstract

Recent years have seen a rise in the popularity of the consumption of sports-related supplements. However, the hypothesis is raised that it is necessary to analyze the quality aspects of these supplements in relation to the information provided on the label, to avoid associated risks and obtain the greatest possible benefit from their consumption. Therefore, the aim of this study has been to carry out an analysis or screening of the protein supplements that are currently marketed in Spain. We analyzed the labels of 52 protein sports supplements available both in physical stores and online. The analysis consisted of addressing three relevant aspects considering the labeling: (a) the legislative framework in which the supplements are marketed, (b) the quality of the protein, and (c) the presence of other ingredients according to the specifications of the label. In the legislative context, there do not seem to be any specific regulations to guarantee consumer protection, which can lead to unfair practices and misleading advertising. Most of the supplements analyzed to comply with the requirements of their current regulations. However, claims about their benefits that are not allowed under European legislation have been found in some of them. Regarding composition and according to label information, the supplements have been found to provide a sufficient dose of protein in terms of recommended protein intake per serving. Regarding the presence of other ingredients and according to the information on the label, most of them, except for egg supplements, contain other ingredients. Colostrum was also found in one of the supplements evaluated. The conclusions of the study reveal that, due to a lack of knowledge or misleading advertising practices, supplements are often not used properly. The information provided is essential for both professionals and consumers to avoid the risks associated with consumption, such as unintentional doping, interactions between ingredients that reduce the quality of the supplement, and consumption of supplements inappropriately, among others.

## 1. Introduction

The consumption of sports supplements is increasingly widespread among the sports community, both elite and amateur. In recent years, both international and national consumption has increased [1]. This increased attention among consumers is due to its nutritional characteristics and its promising ergogenic effects. In the sports context, ergogenic aids are defined as substances used to increase energy, improve performance and prevent nutritional deficiencies, among others [2,3]. Parallel to the increase in the consumption of protein supplements, the ergogenic aid industry has grown exponentially. More and more varieties of protein supplements are becoming available, offering improvements in the composition and new formulations [2].

Protein supplements have proven to be an interesting strategy in the context of sports supplementation. This is due to the multiplicity of benefits associated with its consumption, such as improved body composition, increased muscle mass or hypertrophy, and improved endurance, physical performance, and strength [4,5,6,7,8,9,10,11,12,13]. 

However, both consumers and professionals should be especially careful regarding the use and recommendation of ergogenic aids. Inappropriate use may have associated risks that may compromise consumer health [14]. Risks range from the commercialization of products as ergogenic aids without supporting evidence to unintentional doping, resulting from the involuntary ingestion of a substance banned by the World Anti-Doping Agency (WADA) [6,15,16].

One of the main causes of the inappropriate use of supplements, which will be analyzed in this study, is the absence of specific regulations that regulate the manufacture and marketing of this type of sports supplement. This aspect is striking, considering the existence of extensive legislation in Europe on food marketing. This may be due to the relative novelty in the use of this type of food. However, due to the large increase in their consumption and manufacture that has occurred in Spain in recent years and the potential risks associated with their massive and incorrect use, we believe that it is necessary to review the current legislative framework to promote the safety of the supplement, provide protection against the contamination by doping substances, promote the regulation of labeling, avoid unfair practices and misleading advertising, promote self-control and encourage responsible consumption that always promotes a varied, balanced and healthy diet, as a key element in the athlete’s diet, and the use of protein supplements as a strategy or complement, but never as a substitute for adequate nutrition [6,14,15,16].

Once the legislative aspects have been addressed, the second analysis to be considered is the quality parameters of the supplement itself. In recent years, the amount of protein recommended for different population groups has been studied. Despite the absence of a standardized protocol, there is sufficient evidence to establish consumption recommendations. However, not only the amount of protein is important. Depending on the athlete’s goals, other aspects that determine its quality should be considered: consumption patterns, type of protein, protein dosage in relation to training, and protein composition, among others.

As is well known, one of the parameters that will be used to assess protein quality in terms of supplement composition, and that will be analyzed in this manuscript, will be the presence of branched-chain amino acids (BCAAs) in sufficient quantities. The BCAAs are Leucine, Isoleucine, and Valine, and benefits related to energy metabolism in the muscle are attributed to them [17]. Specifically, leucine has been shown to promote muscle protein synthesis, decrease central fatigue and improve performance [18,19,20,21,22].

In addition to composition, other aspects that have been determined to analyze the quality of the supplement has been to evaluate the intake patterns recommended by the manufacturers and if they are consistent with the existing evidence, depending on the type of protein. Obviously, these recommendations are of a general nature and should be understood in relation to the objectives and needs of each athlete [20,23,24,25,26,27].

As the last aspect of the analysis of protein quality, the digestibility and bioavailability of the protein have been considered according to the information provided by the labeling. For this purpose, various methods have been studied in recent years, such as the biological value of the protein, the net use of proteins, the Protein Digestibility Corrected Amino Acid Score (PDCAAS), or the score of digestible essential amino acids (DIAAS). According to the reviewed literature, none of them have been able to accurately determine protein digestibility and bioavailability, however, the protein biological value has been one of the most successful, and the DIAAS method is proposed as a promising method that may be the reference method in the future, for the calculation of protein digestibility, due to the accuracy of its results. It can be concluded that there is a close relationship between the number of limiting EAA and the quality of the protein in terms of digestibility and bioavailability [28,29,30,31].

The third analysis carried out in this work is about the presence of other ingredients in protein supplements that could increase or decrease their nutritional value. Other macronutrients are often found in their composition, especially when the supplement is marketed for weight gain or post-exercise recovery [32,33,34]. Other common ingredients are those related to performance enhancement. However, although there is evidence to support their consumption, it is important to note that the dosage and pattern of administration may not coincide with the most appropriate protein. They may also interfere with the absorption or bioavailability of the ingredient of interest [35,36,37,38]. 

After all, there seems to be a relatively high risk of finding substances that are not only not beneficial but could be a risk to the consumer. In the context of elite sports, special attention should be paid to substances that are classified as doping, according to the WADA, as their consumption could compromise the athletes’ careers, in addition to the risks associated with their consumption [39]. In addition to banned substances, protein supplements could contain ingredients that, far from being banned, could be indicators of poor quality, such as furosine, which appears, especially in whey proteins, when prepared at high temperatures due to the Maillard reaction [39,40,41,42]. Attention should also be paid to allergens and the presence of enzymes, and in general, to any ingredient that may generate, from absorption problems to the risk of doping. On the other hand, the fact that a supplement does not follow the current regulation cannot guarantee its quality. In addition, unfair commercial practices may be incurred, in which the quality of the protein is not adequate [36,37,38,43,44].

The reason and main objective of this study is the analysis of some protein supplements currently marketed in Spain, according to the information provided by the labeling and based on three aspects: (a) legislative framework, (b) protein quality, and (c) presence of other ingredients. For the evaluation, the classification of the Australian Institute of Sport (AIS) has been followed, which classifies isolated protein supplements according to the protein source: whey, casein, egg albumin, soy, and other plant proteins (hemp, pea, chickpea, rice) [45].

The evaluation of protein supplements and the development of databases are a useful tool for professionals, and this information is essential to ensure the correct nutrition of the athlete. 

## 2. Materials and Methods

### 2.1. Data Collection

For the evaluation of protein supplements, a total of 52 types of protein isolate supplements currently marketed in Spain and manufactured in Europe were listed. They meet the AIS [45] definition of a protein isolate supplement. We selected some of those available both in physical shops and online sales.

Protein isolate supplements show evidence level A (Strong scientific evidence for use in specific situations in sports using evidence-based protocols) according to AIS. For the analysis, the same classification used by the AIS will be followed: whey (dairy), casein (dairy), egg albumin (egg white), soy, and other plant proteins (examples) hemp, pea, chickpea, rice [45]. 

#### Type of Products Tested

- Whey (dairy): It is a protein of high biological value with a high concentration of branched amino acids, including leucine. There are three tips: Concentrated (with 70–80% protein and small amounts of carbohydrates and fats); Isolated (with minimal amounts of carbohydrates and fats and 90% protein); Hydrolyzed (protein chains are broken to give short chain peptides so that their absorption is faster) [45].
- Casein (dairy): It is a protein of high biological value that makes up 80% of milk protein. Due to the acidic environment of the stomach, it forms clots that cause it to be absorbed more slowly [45].
- Egg Albumin (Egg white): It is a protein of high biological value without fat or carbohydrates [45].
- Soy: It is a protein of high biological value; its digestion is fast, and it can be found as isolated or connected. It is usually low in leucine unless it is fortified [45].
- Others plant proteins: It a protein that tends to have lower biological value unless several sources are mixed or fortified [45].

### 2.2. Quality of Data

In order to evaluate the quality of protein isolate supplements, each of them has been subjected to three tests or screens that cover three different aspects (Table 1); (a) Screening 1: legislative framework. In addition, a review of the current legislative framework governing protein supplements has been carried out; (b) Screening 2: protein quality. The rationale and evidence for the use of protein supplements are evaluated; (c) Screening 3: the presence of other ingredients. The ingredients commonly used in the formulation of protein supplements are evaluated.

These three evaluation criteria have been established as determinants of the quality of sports supplements, although always based on the labeling information provided by the consumer and not on analytical data of the product itself. It should be noted that these three evaluation criteria are not currently validated. The evaluation was carried out based on each of the sections, firstly, studying the current legislation that applies to this type of product; secondly, studying the parameters endorsed by the scientific literature that determine protein quality. Thirdly, analyzing the presence of other ingredients, the possible interactions between them, and the possible contamination with doping substances. Legislation relating to labeling and labeling of food supplements, as well as claims and claims contained in protein supplements, has been considered for the analysis of the legislative framework. It will also consider the criteria for nutrition and health claims on foods and the regulation on authorized health claims other than those pertaining to disease prevention and children’s development and health. In addition, the WADA Code covers claims related to other regulations or certifications by private organizations and claims related to other misleading or deceptive advertising [35,39,46,47,48]. 

Protein quality will be evaluated by studying the manufacturer’s recommended dose, the amount of protein per recommended dose, as well as the amount of BCAAs (leucine, isoleucine, valine) and leucine. Further, intake patterns, digestibility, and bioavailability will be examined to determine whether EAA is present or if any limiting is present.

A third screening will examine other ingredients, including macronutrients, performance-enhancing ingredients, ingredients that have no proven impact on the consumer, and allergens [39,49].

It is important to consider that this work does not certify the quality of any supplement, and its publication is for informational purposes, without pretending to advertise any of the supplements analyzed. In addition, it should be noted that a balanced, healthy, and varied diet is the key element in sports nutrition, above any type of supplement, which should always be understood in the context of a strategy to complement the Mediterranean diet. Any type of recommendation for the use of supplements must be supervised by an expert professional and according to the needs and objectives of the supplement consumer.

### 2.3. Database Development

In order to create the database, 52 supplements were compiled, of which: 35 were from whey protein, 5 from casein, 3 from egg albumin, 1 from soy, and 8 from other plant proteins. This database includes the composition of macronutrients and some reference micronutrients in protein-rich foods (Table 2). In addition, the table provides a summary of the analyses carried out for each of the three screenings to provide effective information on different aspects of the quality of the supplement (Table 1).

In order to obtain the information, the labeling of the selected products was analyzed, as well as the manufacturer’s information available on the website, revised at the end of 2021.

The database with the analysis of the supplements studied following the aforementioned methodology will be published. It is also attached to this work as supplementary material. 

### 2.4. Analysis of the Supplements, Attending to the Three Specified Screening

#### 2.4.1. Analysis of First Screening: Legislative Framework

**Figure 1:**
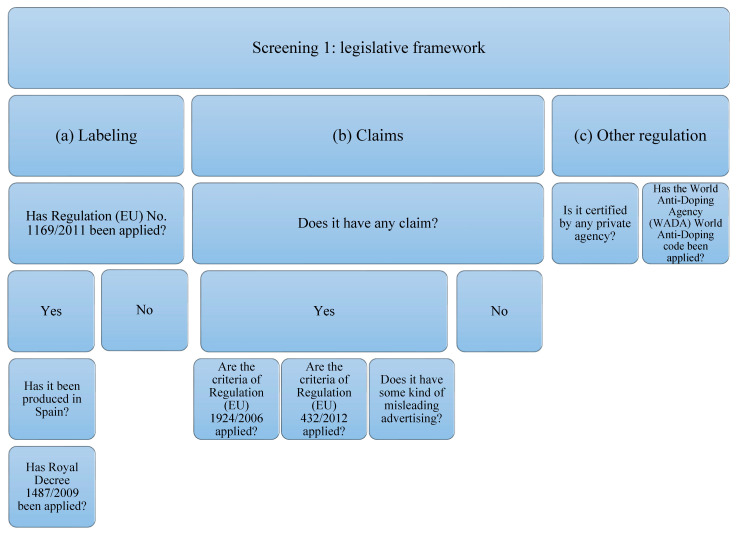
Flowchart Screening 1. The legislation on labeling, claims, and declarations on protein supplements have been taken into account. Claims related to other regulations or certifications by private organizations and the WADA code have also been taken into account.

The supplements studied have been subjected to analysis of compliance with the current legislative framework (Figure 1). In order to carry out the analysis, the legislation that protein supplements must comply with has been taken into account, which, in this case, will be the following: (a) related to labeling: Regulation (EU) No. 1169/2011 [46] on food information provided to the consumer, and, if it has been produced in Spain: Royal Decree 1487/2009 [47] relating to food supplements; (b) that related to the statements and allegations, if they contain them. The criteria of Regulation (EU) 1924/2006 [35] concerning those relating to nutritional claims and health properties in food and European Union Regulation (EU) 432/2012 [48] will be taken into account, which establishes a list of authorized claims of health properties of foods other than those related to the reduction in the risk of disease and to the development and health of children. The presence of any other unauthorized allegation that induces confusion or some type of misleading advertising is also contemplated; (c) that is related to other types of regulation or certification by private organizations and the World Anti-Doping Code of the WADA agency [39]. 

#### 2.4.2. Analysis of Second Screening: Protein Quality

**Figure 2:**
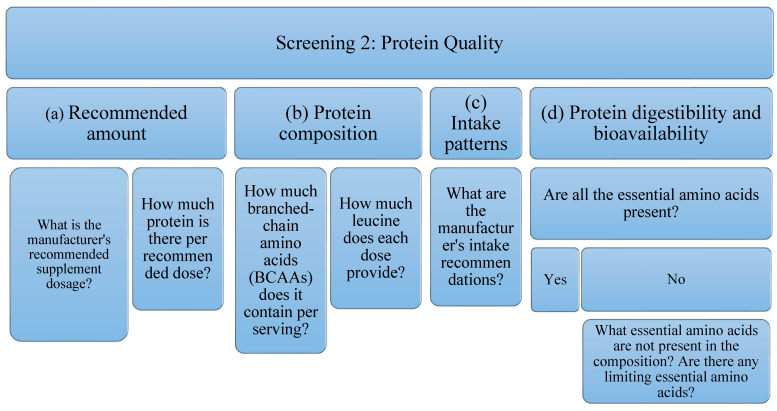
Flowchart Screening 2. It evaluates the manufacturer’s recommended dose, the amount of protein per recommended dose, as well as the amount of BCAAs (leucine, isoleucine, valine) and leucine. In addition, intake patterns, digestibility, and bioavailability are examined.

Due to the high variability of protein supplements, depending on their raw material and their possible uses, it is not possible to establish a filter as a quality criterion for all of them. For this reason, in the present analysis of protein supplements the following aspects will be studied (Figure 2): Related to the amount, (a) the dose recommended by the manufacturer, as well as (b) the amount of protein per recommended dose; This is an interesting piece of information when studying the possible benefit of supplementing the diet with protein supplements; in relation to the composition of the protein and because BCAAs, specifically leucine (in combination with exercise) stimulates protein synthesis by itself, (c) the amount of BCAAs (leucine, isoleucine and valine) has been studied, as well as (d) the amount of leucine present; Regarding intake patterns, (e) the manufacturer’s intake recommendations have also been reflected; being able to study if they are according to the type of protein to obtain its maximum benefits; Finally, with respect to the digestibility and bioavailability of the protein, it will be taken into account if, (f) all the EAA are present, or if, on the contrary, (g) there is some limiting essential amino acid. It is expected that depending on the type of protein (according to its raw material), there will be variability in these data.

#### 2.4.3. Analysis of Third Screening: Presence of Other Ingredients

**Figure 3:**
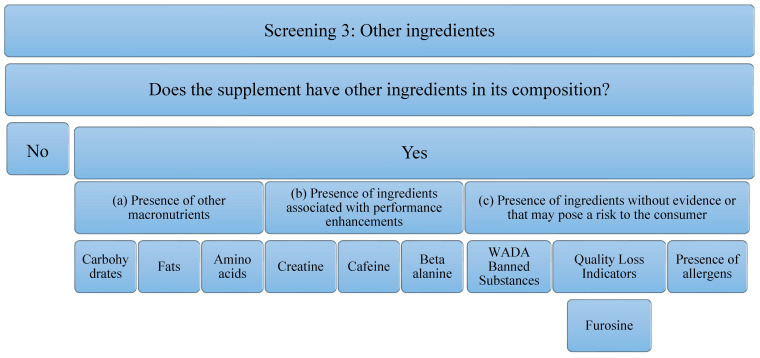
Flowchart Screening 3. Macronutrients, performance-enhancing ingredients, ingredients that have no proven impact on the consumer, and allergens are examined.

Concerning the third screening, the analysis that has been carried out on the supplements studied is in relation to the presence of other ingredients. These ingredients can add value to the supplement or reduce it. The decrease implies, an increase in the economic cost or a decrease in the concentration of the main component of the supplement (in this case, protein), to possible risks to the consumer’s health. It is for this reason that the following aspects will be analyzed *(*Figure 3): (a) the Presence of other macronutrients, understanding that the protein can be pure (100% protein) or have other ingredients such as carbohydrates in its composition, fats, or other free amino acids. In addition to macronutrients, the (b) presence of ingredients associated with performance improvements, such as creatine, caffeine, beta-alanine, has been studied. The presence of these ingredients is important, because, although they have been able to demonstrate evidence in isolated supplementation, it is true that they could negatively interfere with the correct use of the supplement and/or the ingredient of interest (proteins). In addition, the (c) presence of ingredients without evidence or that may pose a risk to the consumer has been studied. Among them, the most important are substances that can lead to involuntary doping, as they are prohibited by WADA [39]. In addition, there are ingredients that could serve as quality indicators when the food has been subjected to industrial processes that may have compromised the nutritional value of the product. An example of this is the presence of furosine, as it was described above. Finally, the presence of allergens must be taken into account, and, above all, the labeling should be developed correctly according to the corresponding regulation [49]. Although these do not pose a risk for all consumers, they must be taken into consideration for those athletes who are susceptible to an allergy or intolerance process. In addition, to eliminate some ingredients that could cause intolerance, formulas with enzymes in their composition have been found. Although they do not pose any risk, they have also been considered in the analysis of this study.

## 3. Results

### 3.1. Results of First Screening: Legislative Framework

A search for various protein supplements on the Spanish market has been developed, and they have been divided according to the criteria established by the AIS into whey (concentrate, isolate, and hydrolysate), casein, egg albumin, soy, and other plants’ protein. Whey-type supplements have mostly been found [45].

In order to check if they comply with current legislation, three regulations have been analyzed: Regulation 1169/2011 [46], Royal Decree 1487/2009 [47], Regulation 1924/2006 [35], Regulation 432/2012 [48] and the indications of the WADA with respect to prohibited ingredients or not recommended.

In relation to Regulation 1169/2011 [46], the main rules that are established to indicate whether or not it is complied with are: if the product name appears, recommended daily dose, ingredients, net quantity (some of the requirements such as storage conditions or warnings, despite that appear in most of them have not been considered to rule them out as valid, since when they are searched online, it is likely that they will appear on the physical product, in addition, the expiration date has not been considered in any of them since they are not it is mandatory to indicate it in the online sale). After analyzing the supplements, it is interesting to note that none of the protein supplements violate these regulations according to the established rules that were taken into account [46]. 

With regard to Royal Decree 1487/2009 [47], the following rules were used to verify whether or not it complied with it: do not state that it prevents, treats, or cures diseases; indicate the recommended daily dose; collect a series of warnings such as, do not exceed the recommended daily dose (despite appearing in some of the products this aspect has not been taken into account because although it does not appear online, it is likely that it does so in the physical product); the information on vitamins and minerals will be expressed as a percentage of the reference values. When analyzing the table, no protein supplement has been found that violates this Royal Decree [47]. 

In order to corroborate Regulation 1924/2006 [35], it was established that it should comply with the following aspects should it make any allegation: It must not be false, ambiguous, or deceitful; encourage excessive consumption of food; claim, suggest, or imply that a balanced diet cannot provide adequate amounts of nutrients; refer to changes in bodily functions that may scare the consumer. In addition, the following claims may be used if the following requirements are met: protein source: only a food may be declared to be a protein source, as well as any other statement that may have the same meaning for the consumer may be made if the proteins contribute at least 12% of the energy value of the food [35]. High in protein: It can only be declared that food has a high protein content, as well as any other declaration that may have the same meaning for the consumer if the proteins contribute at least 20% of the energy value of the food. No supplement has been found that does not comply with the aforementioned aspects, not all of them making claims, although the majority do [35]. 

Concerning Regulation 432/2012 [48], with regard to nutritional claims or claims, it states that as long as they are a “source of protein,” it could be indicated: It contributes to muscle growth; contributes to the maintenance of muscle mass; contributes to the maintenance of normal bones; It is necessary for the normal growth and development of children’s bones. In addition, in relation to the enzyme lactase: improves the digestion of lactose in people with difficulties digesting it (the product must have a minimum of 4500 units). Upon analysis, no supplement has been found to make claims regarding the enzyme lactase. On the other hand, most of the protein supplements made some claim respecting the improvement of muscle mass (especially whey type and proteins of vegetable origin), not all of them being included in the present regulation; Although most were within the legal margin, it should be noted that most of the incorrect claims were made by whey protein supplements [48]. 

Finally, in relation to other regulations, only one supplement has been found that did not comply with the recommendations established by the WADA for containing colostrum since, although it is not strictly prohibited, it is not recommended by it [39].

It is concluded that of the 52 supplements analyzed, all of them seem to be within the framework of the requirements of the regulations. However, it must be considered that there is no specific regulation. With reference to the claims, some whey protein and vegetable protein supplements make claims regarding the improvement of muscle mass without being authorized by the current regulation, for example, declaring that it accelerates the recovery process and prevents muscle catabolism.

### 3.2. Results of Second Screening: Protein Quality

Once the analysis of protein supplements has been carried out, and in relation to the amount of protein recommended by the manufacturer, it is concluded that most manufacturers recommend a serving or contribution per dose of about 30 g of protein, which may vary depending on the brand. Consumed reaching values of 10 or 50 g. With respect to whey protein combinations with other supplements such as collagen, they seem to have a higher amount of protein per serving, reaching values of approximately 40 g. These data seem to comply with some of the recommendations for protein intake per dose (between 20 and 40 g) [20,23,24,50,51]. In addition, no relevant differences have been found in the consumption recommendations between whey protein, egg protein, casein, or in vegetable proteins (soy and other plants), even though the latter may have lower bioavailability. It is for this reason that the AIS [45] recommends increasing the ratio of vegetable protein supplements or fortifying them in those limiting amino acids. In vegetable protein isolates, the highest values regarding the size per dose seem to be found when combining pea and rice protein isolates.

Concerning the composition of the protein, specifically the BCAA content, an estimated content of between 16 and 26 g of BCAAs per 100 g of the product has been observed. There appear to be no relevant differences between whey protein, casein, and whey protein blended with other supplements (whey protein combined). With respect to isolated egg protein, it seems to contain a much lower BCAA value (4.44 g/100 g), although only one of the packages specified the amount of BCAAs contained in the product. Finally, in relation to isolated plant protein, a lower proportion of BCAAs has been observed, between 13–18 g/100 g. The highest values are found in the supplement that combines rice and pea protein isolate, followed by soy protein. Taking into account that most products recommend a serving of 30 g, the amount of BCAAs consumed per serving would be around 4.8–7.8 g/serving in the case of whey protein, casein, and whey protein combinations with other supplements. With respect to plant isolates, it would be found between 3.9–5.4 g of BCAAs per serving.

Despite not having found recommended and protocolized amounts of BCAAs in relation to training, there does seem to be a consensus on AAS in general. Consumption of between 6 and 12 g of EAA is recommended after training [50,52,53,54,55,56,57,58,59,60,61]. In whey and casein protein, BCAA levels are not far below the EAA recommendations, so although no conclusions can be drawn as regards the recommendation for BCAA consumption, it could be suspected that the protein of Whey and casein would be the ones with the highest amounts of BCAAs and are closer to the EAA recommendations. Vegetable proteins would provide lower levels of BCAAs.

Another parameter that has been considered a quality determinant is the presence of leucine in recommended amounts. According to the recommendations, an intake threshold of between 700 and 3000 mg of leucine delivered acutely should be reached [20,50,51]. Of the supplements analyzed, whey protein generally contains between 8 and 10 g per 100 g of protein. No great differences have been found between these and casein or whey protein combinations with other supplements. In relation to isolated egg protein, only one product has been found that details its aminogram, so it cannot be said that it coincides in all cases. The amount of leucine present is 1.95 g/100 g, much lower when compared to the rest of the supplements under study. Finally, isolated proteins of vegetable origin usually contain less leucine, 6–8 g/100 g, the highest values being found in soy isolate and in the combination of rice and pea protein isolate. If one considers that the dose recommended by the manufacturer is usually around 30 g and that in supplements of dairy origin, an average amount of 7.5 g of leucine per 100 g of the product has been found; the dose of leucine per serving would be approximately 2.25 g, that is, 2250 mg of leucine per dose. In the case of vegetable proteins, the dose of leucine can reach 6 g per 100 g of product, thus providing 1800 mg of leucine per dose. In both cases, they comply with the recommended leucine dose. 

Regarding the intake pattern, after analyzing the products under study, it can be concluded that many of the brands do not make intake recommendations, especially in whey protein combinations with other products such as collagen or colostrum, and in isolates of egg protein; In addition, many of the recommendations have been obtained from the website, so it cannot be guaranteed that they will also appear on the physical product. The current scientific evidence does not seem to make it totally clear what your intake pattern should be. However, it seems to be positive in the post-training session; and casein before going to sleep. Perhaps due to the disparity of results in the studies, a wide variety of recommendations can be found depending on the brand and product. Many whey proteins often recommend their product for muscle mass gain and recovery; however, it is also recommended for pre-training, during training, or at any time of the day. There seems to be no differences between the recommendations for the use of whey protein and vegetable protein isolates, with the exception of a vegetable protein isolate that recommends it in the resting phase. Finally, in the case of casein, due to its slow absorption, manufacturers usually recommend its consumption at night before resting [20].

Finally, with reference to the digestibility and bioavailability of proteins, it appears that no type of protein has exhibited limiting amino acids, except for a pea protein isolate where tryptophan was limiting. The rest of the pea isolates, however, did not exhibit any deficiencies in EAAS. In some cases, the aminogram was not provided to the consumer, so it is impossible to determine if the products contained enough BCCA, leucine, or EAA. Animal sources typically have a higher BV than plant sources due to the plant source’s lack of one or more of the EAA [31]. Despite this, the rest of the pea isolates did not present deficiencies in any of the EAAS. It seems interesting to note that some of the products studied did not disclose the aminogram to the consumer, so it is impossible to conclude whether they contain enough BCCAs, leucine, or EAA. 

These things considered, it should not be forgotten that the use of any supplement must be performed under the direction of a nutrition professional and must be evaluated in the context of a diet adapted and personalized to the athlete’s circumstances.

### 3.3. Results of Third Screening: Presence of Other Ingredients

With regard to the presence of other ingredients and after analyzing the different protein supplements, it has been observed that most of them contain other ingredients in addition to the protein hydrolysate. In the case of whey protein, it usually contains sweeteners, the most common being sucralose, and emulsifiers such as soy lecithin are added to some of them; In addition, most contain aromas since they usually give them different flavors, one of the most common being the chocolate flavor, these also usually contain cocoa; on the other hand, some of them also have enzymes added. No major differences have been observed between whey protein supplements and those of casein or whey protein combined with other supplements (in this case, the combined ones contained whey protein and collagen or colostrum), except that the latter two are not usually added enzymes, only a combination of whey protein and colostrum has been found to which enzymes were added. Regarding plant protein isolates, they do not usually contain enzymes, but other ingredients such as B12 or certain components that consumers could identify as advantageous are usually added to them, for example, certain vitamins (vitamin C, A…), quinoa; only one fortified with BCAAs has been found to improve the aminogram. Finally, in the case of egg protein isolates, none have been found with additional ingredients in addition to the isolated protein. Claims on ingredients such as the presence of vitamins such as vitamin B12 or B6 are allowed, as long as it meets the specifications of Regulation (EC) 1924/2006 [35]. 

About the ingredients associated with benefits in athletic performance, no supplements have been found to which ingredients are added with evidence A according to the AIS; however, several whey protein supplements add collagen to their products, and one of the plant isolates fortifies its product with BCAAs, with evidence B and C according to the AIS respectively. In addition, certain plant protein isolates add vitamin C, the evidence of which is B [62].

No supplements have been found to which ingredients prohibited by WADA are added. However, one of the supplements analyzed contained colostrum, which is discouraged by both the WADA and the AIS since it is possible that they contain IGF-1 growth factors, among others, which are prohibited and could lead to positives in anti-doping tests [39]. No furosine was found in the composition of any of the supplements analyzed, an indicator of loss of quality in nutrients due to subjecting the supplement to high temperatures.

As regards the presence of allergens, it has been observed that all whey protein supplements contained the allergen “dairy,” most had “soy,” and very few contained “lactose” (dairy); some also had other allergens such as “nuts” or “eggs.” Not all products to which enzymes had not been added contained lactose, although some of them indicated that it could facilitate their digestion, possibly in case there were traces of this or other sugars after hydrolysis. No differences have been found between allergens present in casein and whey protein blended with other supplements, and whey protein. On the other hand, in relation to isolated egg protein, most contain egg as the only allergen; however, one of them pointed out that it may contain traces of “dairy,” “soy” and “gluten.” Finally, certain plant protein isolates indicate that they could contain “oats,” “wheat,” “dairy,” or “gluten,” among others; however, some of them claim to have no allergens.

Some of the whey supplements declared the absence of “lactose” in their composition. Many of the whey proteins contain digestive enzymes, such as cellulase, amylase, lactase, lipase, and protease; some also add beta-D-galactosidase. Since not all supplements to which enzymes were added claimed to be lactose-free and by adding several enzymes in addition to lactase, it does not appear that these enzymes are added specifically for lactose, although it could be a factor. The enzyme lactase improves the digestion of lactose in people with problems digesting it. It can only be used in the case that the food supplement contains a minimum amount of 4500 units of the FCC (Code of Chemical Substances in Food), also indicating that it must be consumed with foods that contain lactose [63]. Lastly, no specifications have been found for lactose content; it is simply reflected in the allergens if they can contain them. Other proteins, such as vegetable proteins, claim the absence of lactose, so that, due to the type of composition of the product (vegetable sources), it serves to add value to the product.

## 4. Discussion

### 4.1. Discussion of First Screening: Legislative Framework

#### Current Legislative Framework Regulating Protein Supplements

There is currently no specific regulation at the European level regulating ergogenic nutritional aids. In 2001, the Directorate-General for Health and Consumer Protection of the European Commission requested the Scientific Committee for Food (SCF) the need to draw up a specific report on the specification of sports food. In this document, it was concluded that a well-balanced diet is a basis for a correct diet in the athlete. However, in the same document, it is established four categories of products: (a) Foods and beverages rich in carbohydrates (HC); (b) HC and electrolyte solutions; (c) Foods and beverages rich in proteins (in whose definition it established that the minimum composition of proteins in protein concentrates should be equal to or greater than 70%); (d) Supplements and other food components. It was not until 2008 that the European Commission began to consider the possibility of regulating these products solely through the health claims authorized in Regulation (EC) No. 1924/2006 [35]. In implementing the regulation, the European Food Safety Authority (EFSA) issued favorable declarations on foods used in physical effort, supported by the European Commission and the Member States, and incorporated in the Annex to Regulation (EU) 432/2012 [48], which establishes a list of authorized claims of health properties of foods other than those related to the reduction in the risk of disease and to the development and health of children; however, none of these statements refers to the specific consumption of protein supplements. Regulation (EC) No. 1924/2006 [35] provides the legal framework and rules for requesting the evaluation of these statements. To conclude, foods not included in Regulation (EU) No. 609/2013 [46] in foods intended for infants and young children, foods for special medical purposes, and substitutes for the complete diet for control weight, will be regulated by the legal aspects applicable to all foods [5,6,14,16].

In the specific case of Spain, only products presented in the form of food supplements are required to be notified according to Royal Decree 1487/2009, of 26 September, regarding the food supplements [47]. Those nutritional supplements that do not meet the requirements of food supplements (especially those referring to the form of marketing) will not be food supplements, just “naturally rich foods” or “fortified foods.” In addition, “the requirements of the royal decree shall not apply to food supplements legally manufactured or marketed in other member states of the European Union, nor to products originating in the countries of the European Free Trade Association (EFTA) that are contracting parties in the Agreement on the European Economic Area (EEA), nor to the States that have a Customs Association agreement with the European Union” [5,47].

There is no specific legislation for this type of supplement that contributes to increasing the risks associated with their consumption. In addition, it promotes misleading advertising, such as those related to the supposed efficacy of the product, without any scientific evidence [5,14]. 

Despite the absence of specific regulations, many companies that produce protein supplements “self-regulate” and claim certification through quality audits, carried out on a voluntary basis and take into account the list of prohibited substances issued by the WADA and its World Anti-Doping Code should be taken into account [39]; However, this information is not considered in current European legislation, in fact, WADA warns that many of the dietary supplements marketed may contain substances that could give positive results in anti-doping controls [15,64,65].

### 4.2. Discussion of Second Screening: Protein Quality

#### Rationale and Evidence for the Use of Protein Supplements

In the context of protein needs, there is some controversy concerning consumption recommendations, as these will largely depend on the level and type of physical activity, among other factors such as recent exercise stimulus [5,20,66]. In healthy individuals, the dynamic balance between protein degradation and synthesis ensures the maintenance of muscle protein. The availability of nutrients and physical exercise are the greatest stimulants of protein synthesis. Therefore, it is necessary to ensure an adequate supply of this macronutrient, as well as its quality [22]. 

Evidence supports the fact that adequate protein intake contributes to optimal health, improves athletic performance, and prevents age-related loss of muscle mass (sarcopenia) and excessive wear and tear. In addition, it has an effect on satiety and weight control [4,20,66,67]. 

However, when it comes to adequate consumption, it is not only about (a) quantity but also about quality in protein replacement. In this context, it is important to take into account aspects such as (b) protein quality in relation to amino acid composition, as well as aspects related to (c) intake patterns throughout the day and the dose per snack and (d) protein digestibility [66]. 

All these aspects must be understood in the context of a diet in accordance with the needs and targets of the athlete. It must be understood that the diet is sufficient to cover protein requirements, and supplementation is a complement and not a substitute for a varied, balanced, and healthy diet.

- Recommended amount

Despite the absence of a standardized consumption protocol, the International Olympic Committee, as well as the International Society of Sports Nutrition, recommend the combination of resistance exercise and the daily consumption of 1.6–2.2 g/kg of body mass/day of protein. In this sense, this value is in terms of total protein consumption per day [20,50,51].

- Protein composition

Amino acids represent the basic units of proteins, but they do not all have the same properties. For this reason, not only does the amount of protein stand out but also its quality [31]. One of the main determinants of protein quality is the presence of EAA. Despite the absence of established protocols, many studies support the idea that ingestion of between 6 and 12 g of EAA after resistance exercise stimulates protein synthesis. Although more research is needed on this, it is clear that ingesting quality protein sources can enhance training adaptations [50,52,53,54,55,56,57,58,59,60,61]. 

EAAs are comprised of nine amino acids, some of which have received special attention due to their predominant role in muscle protein synthesis [68,69]. As is known in the literature, the amino acids considered BCAAs, leucine, isoleucine, and valine represent 50% of the EAA, so they must be supplied exogenously through the diet [22].

It has been proven that the isolated contribution of BCAA in the fasting state optimizes the synthesis of muscle proteins. However, although at the plasma level it increases the levels of BCAA, the balance between synthesis and degradation remains negative, so the BCAA intake independently appears not to stimulate an anabolic state. However, it must be taken into consideration that the activation of the anabolic pathway and the increase in muscle protein synthesis could act through independent mechanisms. That is, the increase in insulin levels activates the anabolic signaling pathway but it is not associated with improved muscle protein synthesis in the absence of EAA [21,22]. 

Supporting the aforementioned, Rabassa-Blanco et al. published a review on the effect of protein and BCAA supplements on changes in body composition, muscle mass, central or peripheral fatigue, muscle strength, muscle damage, and anabolic response in muscle recovery. They showed that protein supplements, in association with a balanced diet and training, can improve performance and muscle mass [19]. Despite the number of studies on BCAAs, there seems to be no consensus on the recommended amount per dose in relation to training. Of all the BCAAs, specifically, leucine has shown positive effects on muscle mass, in reducing central fatigue and consequently improving performance [19,70,71]. 

Another study published by The American Journal of Clinical Nutrition [18] assesses the combination in the consumption of protein in different doses together with different amounts of leucine, as well as the improvement in the synthesis of muscle protein associated with this consumption. The results show that the addition of a higher dose of leucine to a smaller amount of protein (6.25 g) improved the myofibrillar protein synthesis rate (MPS) to the same level as that observed with four times more protein of serum (25 g). That is, suboptimal protein doses may be more effective in stimulating MPS by adding a high proportion of free leucine [18]. Although more work is needed on this idea, in addition to protein intake, evidence supports an acute intake of 700–3000 mg of leucine, in addition to a balanced range of EAA [20,50,51].

- Intake patterns

As seen previously, the beneficial effects on hypertrophy and performance related to protein consumption are enhanced if its consumption is associated with moderate training and in the context of a healthy diet. In this way, the importance of adequate planning of protein intake is emphasized, not so much of the daily amount, but of the ideal time of consumption to obtain the greatest effect.

On the other hand, it is important to highlight the importance of adequate distribution of each of the day’s snacks and that these should be in accordance with physical activity. Research points to continued consumption during the day, avoiding long periods of protein fasting. The practice of physical exercise, especially strength, associated with the consumption of protein, maximizes protein synthesis for approximately 24 h after exercise. This is due to the increased sensitivity of muscle tissue to amino acids. However, it depends on the composition of these proteins [20,50]. There is evidence that the protein dose should ideally be administered every 3–4 h throughout the day. However, individual tolerance aspects must be considered and especially consider that the greatest beneficial effect derives from ingestion before or after training; Although the anabolic effect of exercise appears to be long-lasting (about 24 h), it probably decreases with increasing time after exercise. A dose should be included after exercise and another one close to bedtime in order to maximize the stimulus of muscle synthesis and thereby improve performance, and always in combination with training and a balanced diet [20,50,51,67].

Associated with the aforementioned, the concept of “protein stimulation” arises, which seems to have a close relationship with cardiometabolic and body composition benefits when combined with multimode or multicomponent exercises (resistance, interval, stretching, endurance). The goal is to provide a continuous protein intake of between 5–6 meals a day with an intake of 2.0 g per kilogram of bodyweight per day. This supposes a contribution of between 20 and 40 g of protein per dose [23,24].

In 2017 the Academy of Nutrition and Dietetics, Dietitians of Canada, and the American College of Sports Medicine published their opinion on protein consumption in relation to metabolic responses. They show a grade I (good) protein intake (approximately 20–30 g of total protein, or 10 g of EAA) during exercise or in the recovery period. This consumption recommendation promotes an increase in protein synthesis in the muscles and a better nitrogen balance [4]. 

Other studies support the idea of an intake of 10 g of EAA within 2 h of exercise to improve muscle protein synthesis or a total protein intake of 0.25 to 0.3 g/kg body weight or 15 to 25 g protein with carbohydrates as soon as possible after exercise, but larger amounts may be warranted based on body composition and training cycle [59,60,61]. 

Finally, although bioavailability and digestibility will be discussed later, it must be considered that different types of protein will have different absorption. For this reason, intake patterns will be determined according to the type of protein, among others.

For example, casein, which is slowly absorbed, consumed before sleep (about 30 min before and two hours after dinner), and in an amount of approximately 30–40 g, appears to increase muscle protein synthesis without affecting lipolysis and fat oxidation at night [20,23,24,25,26,27]. When casein is consumed, it forms clots in the acidic environment of the stomach, which slows down digestion and the supply of amino acids to the body. This fact was verified by measuring the respiratory quotient (CR) in the morning after the ingestion of casein, in comparison with placebo and other proteins. It was found that CR levels in the group that consumed casein were maintained [72]. Furthermore, it does not seem to increase insulin and hunger in the morning either [73].

- Protein digestibility and bioavailability

In addition to the factors considered above, important aspects that influence the digestion of proteins and the interaction with other foods must be taken into account. That is to say, factors such as the composition of the protein, the dose, and the timing of ingestion, will be determinants of the maximum potential quality, which largely depends on whether it reaches the site of action in an adequate quantity after ingestion [31]. 

The biological value (BV) of the protein will be one of the key aspects. The BV provides a measure of how effectively the body uses proteins ingested through the diet. It is calculated by dividing the nitrogen used for tissue formation by the nitrogen absorbed from food. It is multiplied by 100 and is expressed as the percentage of nitrogen used. In general terms, food with high BV is correlated with a high supply of EAA [31]. 

Another parameter used in the measurement of digestibility and bioavailability is the net use of proteins. It is the same as the BV of proteins with the exception that, instead of being calculated with absorbed nitrogen, it measures the ingested nitrogen. That is, it takes into account the relationship between what is ingested and what is used for tissue formation, taking into account the nitrogen that is not used from the food itself [31]. 

On the other hand, the Protein Digestibility Corrected Amino Acid Score (PDCAAS) measures the quality of the protein as a function of the content of the first limiting EAA, as a percentage of the content of a reference standard of EAA. That is, it is the ratio between the first limiting amino acid in a gram of the protein to be studied and the value of the reference protein or required value. This value is multiplied by the digestibility of the protein. Fecal digestibility is the difference between what is digested and what is excreted. However, the amino acids that reach the terminal ileum can be consumed by the bacterial flora without being used by the body, although they do not appear in the feces. On the other hand, antinutritional factors such as trypsin inhibitors, tannins, and lectins, present in some protein-rich foods such as soybean meal, peas, and beans, could increase protein losses in the terminal ileum, reducing protein hydrolysis and the absorption of amino acids. A more precise determination is proposed taking into account ileal digestibility [28,29].

Although this measure has been used for twenty years and has been very useful, its limitations have been recognized and should be reassessed. WHO/FAO recommends that amino acids in the diet be considered as individual nutrients, as PDCAAS could underestimate quality [28,29,30]. 

In 2011, FAO announced that the PDCAAS method contained limitations, and in 2013 a recommendation for the use of the DIAAS method was published: score of digestible EAA, which is% DIAAS = 100 × [mg of DIAAS of the diet in 1 g of dietary protein/(mg of the same dietary amino acid in 1 g of reference protein)] [28,30]. In order to perform this calculation, digestibility should be based on the actual ileal digestibility of each amino acid, preferably determined in humans (although it is also calculated in pigs and rats). Likewise, for the calculation, the recommended amino acid scoring patterns, or amino acid patterns of the reference protein for three age groups (infants, young children up to 3 years, and older children, adolescents, and adults) must be taken into account.

It could be concluded that according to the DIAAS analysis, a protein will be of higher quality the lower the amount of limiting amino acids (the one that is not found in food), that is, the lower the deficit of EAA, since that to achieve optimal protein synthesis it is necessary that EAA are present [74]. The application of the DIAAS is a hopeful method published in 2013 by FAO. However, it is still necessary to carry out more reviews and research to be able to be used as a reference method in the calculation of protein digestibility [74]. 

Until that moment, the BV seems to be one of the most reliable measures for calculating the protein quality of foods. However, it should be considered that it measures maximum protein quality potentials, but this will vary greatly according to individual parameters [31]. In general, all the methods agree that the proteins used to make protein supplements have high-quality indices in terms of digestibility and bioavailability, especially those from dairy sources.

### 4.3. Discussion of Third Screening: Presence of Other Ingredients

#### Ingredients Commonly Used in the Formulation of Protein Supplements

- Presence of other macronutrients

It is common to find other ingredients in protein supplement formulas. These ingredients can be based on scientific evidence, for example, as classified by the Australian Institute of Sport or not be associated with any supporting evidence and even pose a risk to the consumer [45]. 

First, it is important to know that protein supplements can be found in isolation, simply providing proteins (from one or more protein sources, as a mixture of proteins); or with the addition of some ingredients. The most common are carbohydrates, which are mainly found in recovery formulas after exercise. Carbohydrates are essential for the replacement of glycogen after sports practice since, in most cases, they are the main source of energy that the body demands during exercise. Adequate replacement of these ensures adequate recovery [32,33,34]. 

There is some controversy in relation to the so-called “anabolic window,” in which the body is more susceptible to recovery if the intake is carried out immediately after the end of the exercise session. However, the evidence suggests that the muscle glycogen resynthesis process begins immediately afterwards, with the highest resynthesis rate being between 30 and 60 min after sports practice. This fact is due to an increase in the translocation of the glucose transporter protein (GLUT-4) due to an increase in the concentration of calcium associated with exercise. In addition, low levels of glycogen lead to an increase in the activity of glycogen synthase, an enzyme responsible for the synthesis of glycogen [32,33,34,75,76,77]. Concerning the recovery of glycogen, protein intake takes on a leading role. There seems to be a synergistic action of carbohydrates + protein associated with the stimulatory effect of insulin secretion. The type of protein also influences its insulinotropic action, those hydrolyzed that exert the greatest effect. Likewise, whey protein has reported higher activity, possibly due to its high leucine content, than casein [78]. 

On the other hand, fats can also be found in the composition of protein supplements, although these are less common. They are associated with formulas for weight gain or volume, providing a high-energy supplement [45]. However, it should be taken into account that supplements enriched with other macronutrients, such as carbohydrates or fats, will have reduced protein content, which may influence the expected results of the supplement. In addition, it must be considered that not all nutrients are necessary for all training sessions and that poor management of these can interfere with the athlete’s goals. For example, interfering with energy balance and nutrient density, stimulating satiety, absorption of nutrients, generating gastrointestinal problems, and even increasing the risk of allergies and intolerances [45].

Another ingredient commonly found in protein supplements is individual amino acids [45]. That is, sometimes, it is interesting to fortify or enrich protein supplements from sources with limiting amino acids, such as leucine, in supplements from plant sources [79,80,81].

- Presence of ingredients associated with performance improvements

These include the addition of creatine, caffeine, beta-alanine, among others. While it is true that these ingredients may have shown efficacy in sports supplementation, their use protocols must be respected. The recommendations associated with the consumption of protein supplements may not be the same as those of the added ingredients. It is also possible that the doses of these ingredients in the protein supplement are not optimal. For this reason, it is preferable that these ingredients are consumed as individual products so that the athlete maintains control over when and how they are used [45]. However, it is a field that should continue to be investigated, with the aim of enhancing the athlete’s goals by consuming isolated protein supplements or those with multiple ingredients [36,37].

- The presence of ingredients without evidence or that may pose a risk to the consumer

The AIS recommends avoiding the consumption of multi-ingredient supplements, as they are more expensive and also increase the potential for doping or contamination [45]. 

Among the main risks are those related to a greater difficulty of absorption or even interaction in the absorption of the ingredients of interest (proteins or amino acids, in this case), gastrointestinal problems associated with their consumption in the context of sports practice, increase in gastric emptying, increased cost of the supplement, decreased efficacy, risk of doping, among others [38,43,44].

Another interesting ingredient, which could serve as a quality indicator, is the presence of furosine. Furosine is a molecule that appears when enzymatic browning reactions occur (Maillard reaction) by subjecting a reducing sugar and an amino group to high temperatures (for example, subjecting whey protein to the spray-dryer technique). In certain foods, these reactions are intentional; However, in protein products, they could assume that the product has been treated at high temperatures, which could have produced damage to the amino acids and, therefore, affected the nutritional value of the supplement [40,41,82]. In addition, it should be noted that there seems to be a relationship between lactose content and greater heat damage [82]. 

Another risk associated with the presence of more ingredients is that it increases the risk of allergens, and some of the most common allergens will be dairy in whey and casein proteins, eggs in egg albumin, and finally, soy and nuts in vegetable proteins [49]. 

Regarding dairy products, it is worth noting the presence of food enzymes in many of the supplements, with the aim of reducing or eliminating the amounts of lactose. In addition, according to the protein purification or extraction process itself, the content of other ingredients is eliminated, such as lactose; thus, isolated protein (90% protein) has a lower lactose content than concentrated protein (70–80% protein). Until now, there has been no explicit regulation on the lactose content of food. However, the EFSA developed a scientific opinion where it establishes lactose thresholds in lactose intolerances and galactosemia. The panel notes that most individuals diagnosed with lactose intolerance or lactose maldigestion can tolerate 12 g in a single dose. In addition, in a non-binding manner, the EFSA establishes guidelines about labeling: “Lactose-free” food products are those that certify a percentage of less than 0.01% lactose; while “low lactose content” will be for those with lactose content lower than 1% [83].

## 5. Conclusions

The use of protein supplements is becoming increasingly popular in the context of sports context, and their marketing has increased in recent years. However, it is necessary to carry out quality screening according to the labeling information provided by the manufacturer. To carry out this research work, 52 protein sports supplements currently commercialized in the Spanish market were analyzed, and a database was created as complementary material. Concerning Screening 1, in the legislative framework, the analyzed supplements seem to meet the requirements of the regulations that regulate them. However, it should be noted that, in the absence of specific regulations for this type of supplement, the legal margins are wide and not very specific. In addition, many of the whey protein and vegetable protein supplements make claims regarding the enhancement of muscle mass without this being authorized by current regulations.

Regarding Screening 2, protein quality, it seems that the supplements provide a sufficient dose of protein in terms of recommended protein intake per serving. Even though vegetable proteins may lack some amino acids and have a lower bioavailability, it is recommended to fortify them with those limiting amino acids. With respect to the presence of BCAAs, it seems that the highest content is found in whey protein and casein, as opposed to vegetable proteins and, lastly, egg protein. The first two are in line with current BCAA intake recommendations. There appear to be no specific recommendations on intake patterns except for protein isolate, which is recommended at rest, and casein, recommended before bed. Finally, according to the studied aminograms, no protein presented any limiting amino acid, although animal proteins usually have a higher biological value than vegetable ones.

Lastly, about Screening 3, which studies the presence of other ingredients, it seems that most of them, except for egg ingredients, contain other ingredients in their composition, many of them considered advantageous, and of which, in addition, claims are permitted if they comply with the regulations. No WADA-banned ingredients have been found, except for the colostrum in one of the supplements, which is discouraged. Ingredients indicating quality loss are not present either, although some allergens such as dairy, soy, egg, and lactose are present. Some of them declared the absence of lactose, although the presence of enzymes does not seem to correlate with it. It must be considered that the analysis has been carried out based on the labeling and not on the product itself, so it would be advisable in the future to analyze the veracity of the labeling experimentally.

Finally, it should be noted that, although protein supplements are an adequate tool in the athlete’s diet, it is necessary to consider certain aspects, such as those analyzed in this research work, before giving any recommendation by professionals. In addition, supplementation must always be understood in the context of a healthy, varied, and balanced diet and never pose a risk to the consumer, who must be informed and advised correctly in relation to his objectives and needs. This work aims to shed light on current knowledge of sports supplementation and certify consumer protection against unfair practices, as well as ensure the quality of the supplement.

## 6. Limitations and Future Research Paths

It is important to note that despite the recommendations, each individual situation is different, and there are many factors that influence such as the amount, intensity, and type of exercise, the age of the athlete, the time they exercise, and their objectives; For this reason, adequate personalized planning is necessary, carried out by an expert professional in the area.

The analysis relating to the legislative framework, protein quality, and presence of other ingredients has been carried out on the basis of the information provided to the consumer and not by analyzing the supplement itself. However, the practical implications of the results of this study include the development of a database of protein supplements that provides information for each of the three screenings in order to provide effective information on different aspects of supplement quality. For future research, it is suggested to test the quality of these supplements, analyze the content and verify if there is any difference between the information provided and the actual content, such as contamination with doping substances, protein content total, or presence of other undeclared ingredients.

## Figures and Tables

**Table 1:** Screening components are included in the supplement database.

Screening 1: Legislative Framework	
Labeling	Regulation (EU) No. 1169/2011
	If it has been manufactured in Spain: Royal Decree 1487/2009
Claims	Regulation (EU) No. 1924/2006
	Regulation (EU) 432/2012
	Other type of advertising outside the legislative framework
Other regulation	Other certifications
	World Anti-Doping Agency (WADA)
**Screening 2: Protein quality**	
Recommended amount	Manufacturer’s Recommended Dose
	Amount of protein per recommended dose
Protein composition	Amount of branched-chain amino acids (BCAAs) (leucine, isoleucine, and valine)
	Amount of leucine present
Intake patterns	Manufacturer’s intake recommendations (intake patterns)
Protein digestibility and bioavailability	Presence of essential amino acids. (EAA)
	EAA not present (limiting essential amino acid)
**Screening 3: Presence of other ingredients**	
Presence of other macronutrients	Presence of carbohydrates, fats, and other amino acids in free form
Presence of ingredients associated with performance enhancements	Presence of ingredients associated with performance enhancements such as creatine, caffeine, beta-alanine
Presence of ingredients without evidence or that may pose a risk to the consumer	WADA Prohibited Substances
	Quality loss indicators
	Presence of allergens

**Table 2:** Components included in the composition of protein isolate supplements database and how they are abbreviated.

	Product (100 g)	Commercial House	Kilocalories (Kcal)	Protein (g)	Aminogram (g)			Branched-Chain Amino Acids (BCAA) (g)	Carbohydrates (g)	Of Which Sugars (g)	Fats (g)	Sodium (mg)
**Abbreviation**	PR	CH	KCAL	PROT	AA			BCAA	HC	SUG	FAT	NA
	Screening 1								Screening 2			
	Regulation 1169/2011	Royal Decree 1487/2009	Regulation 432/2012	Regulation 1924/2006	World Anti-Doping Code (World Anti-Doping Agency)				Amount of protein per recommended dose	Limiting amino acid		
**Abbreviation**	R1	R2	R3	R4	R5				AP	LA		
									Screening 3			
	Others manufacture’s intake recommendation	Allergens	Enzymes	Additives	B12 (mi-crograms)	Serving	Degree of evidence AIS	Certification	Frequency	Source of information	Complies with what is established in the legislation	
**Abbreviation**	OR	AG	EZY	ADD	B12	SG	DE	CT	FC	SI	√	

## Data Availability

The data used to support the findings of this study can be made available by the corresponding author upon request.

## Supplementary Materials

The following supporting information can be downloaded at: https://www.mdpi.com/article/10.3390/foods11213500/s1, Analysis and screening of commercialized protein supplements for sports practice.
