# Supplementary material for: Analysis and Screening of Commercialized Protein Supplements for Sports Practice
Source: Foods. 2022 Nov 3;11(21):3500. doi: 10.3390/foods11213500 (PMC9658000; doi:10.3390/foods11213500)
Supplement: Supplementary file 1 [file foods-11-03500-s001.zip › foods-1968180-supplementary.pdf]

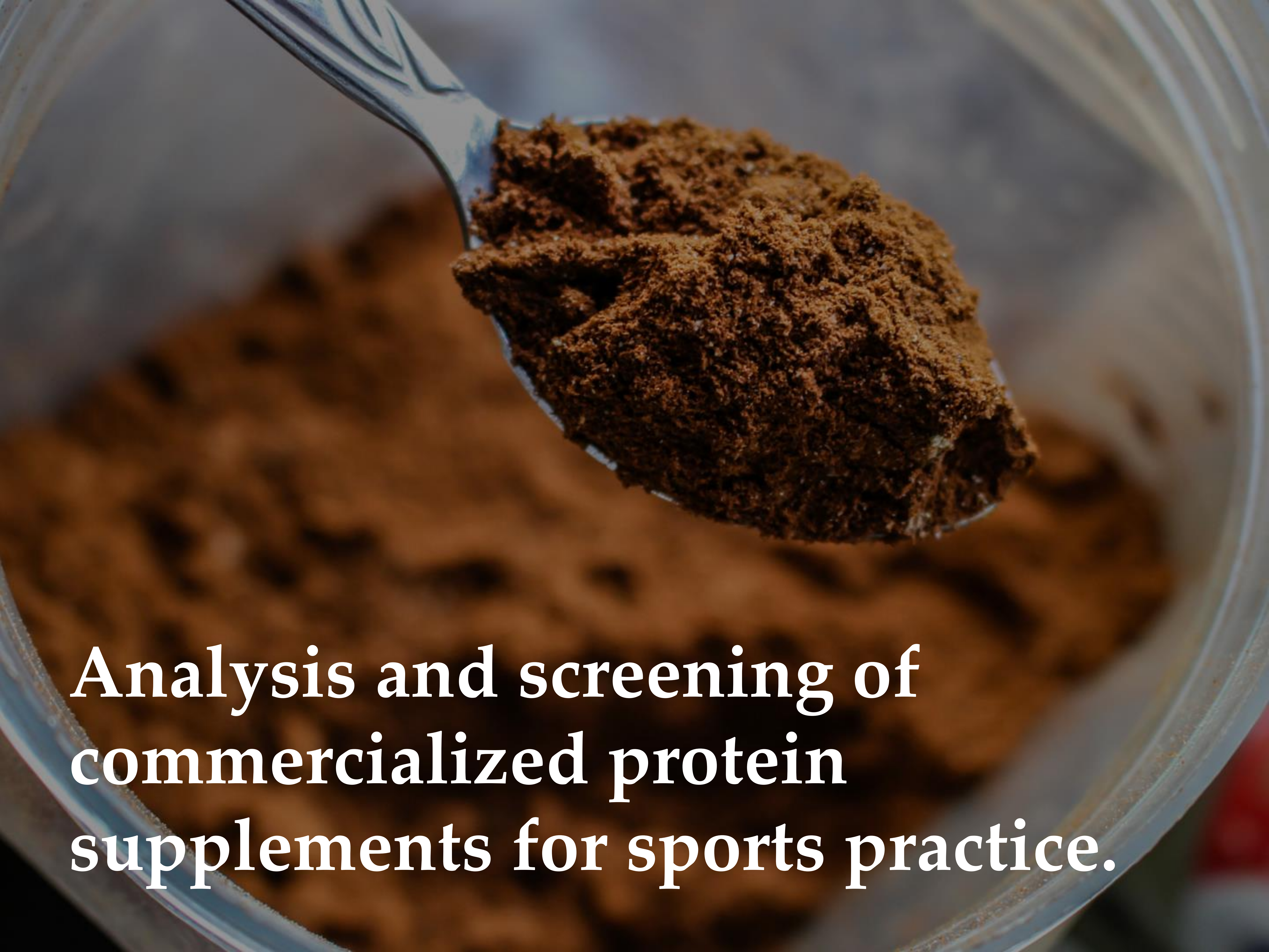

Analysis and screening of commercialized protein supplements for sports practice.

Analysis and screening of commercialized protein supplements for sports practice.

- **Paloma Rodriguez-Lopez[1], Ascensión Rueda-Robles[1*], Leticia Sánchez-Rodríguez[1], Rosa María Blanca-Herrera[1], Rosa María Quirantes-Piné[2], Isabel Borrás-Linares[2,3*], Antonio Segura-Carretero3+, Jesús Lozano-Sánchez[1+]**

[1] Department of Food Science and Nutrition, University of Granada, Campus Universitario s/n, 18071-Granada, Spain; palomarlopez@ugr.es; ruedarobles@ugr.es; rblanca@ugr.es; jesusls@ugr.es

[2] Research and Development Functional Food Centre (CIDAF), Health Science Technological Park, Avenida del Conocimiento 37, Edificio BioRegión, 18016 Granada, Spain; rquirantes@cidaf.es; iborras@cidaf.es.

[3] Department of Analytical Chemistry, Faculty of Sciences, University of Granada, Granada 18071, Spain; ansegura@ugr.es

* Corresponding author:
E-mail address: iborras@ugr.es; ruedarobles@ugr.es
Tel.: +34958637083
+These authors are joint senior authors on this work

The consumption of supplements associated with sports practice has increased in popularity in recent years. Among the most commercialized are protein supplements. However, there is much confusion regarding the safety of its consumption, as well as in reference to the terms of standardized intake protocols with a view to obtaining the greatest benefits. In the legislative context, there does not seem to be a specific regulation that guarantees consumer protection. This can lead to unfair practices and misleading advertising. On the other hand, it is essential to analyze aspects of the quality of the supplement, since, beyond protein content, it is important to cover aspects of composition, intake patterns and aspects of digestibility and bioavailability. Finally, the presence of other ingredients in the composition must be considered, such as those with evidence-based ergogenic effects or those without evidence that may influence the quality of the supplement or even represent a risk to the consumer. The present work aims to perform an analysis or screening of the aspects of protein supplements currently marketed in Spain. The supplements will be subjected to three screens: a) legislative framework; b) protein quality and c) other ingredients. As a result, a database of protein supplements, once analyzed, is published. The objective is that both professionals and consumers have a key tool for the responsible use of protein supplements, in the context of a varied, balanced, and healthy diet. This information is essential to guarantee a correct diet in the athlete and reduce the risks associated with the consumption of sports supplements.

INDEX

**Whey (dairy)**	**It is a protein of high biological value with a high concentration of branched amino acids, including leucine. There are three tips: Concentrated (with 70-80% protein and small amounts of carbohydrates and fats); Isolated (with minimal amounts of carbohydrates and fats and 90% protein); Hydrolyzed (protein chains are broken to give short chain peptides, so that their absorption is faster).**
Casein (dairy)	It is a protein of high biological value that makes up 80% of milk protein. Due to the acidic environment of the stomach, it forms clots that cause it to be absorbed more slowly.
Egg Albumin (Egg White)	It is a protein of high biological value without fat or carbohydrates.
Soy	It is a protein of high biological value; its digestion is fast, and it can be found as isolated or connected. It is usually low in leucine, unless it is fortified.
Others plant proteins	**It is a protein that tends to have lower biological value unless several sources are mixed or fortified.**

Abbreviations

Product (100 grams)	Commercial house	Kilocalories (Kilocalories)	Protein (grams)	Aminogram (grams)	BCAA (grams)	Carbohydrates (grams)	Of which sugars (grams)	Fats (grams)	Sodium (milligrams)
PR	CH	KCAL	PROT	AA	BCAA	HC	SUG	FAT	NA

**Screening 1**					**Screening 2**	
Regulation 1169/2011	Royal Decree 1487/2009	Regulation 432/2012	Regulation 1924/2006	World Anti-Doping Code	Amount of protein per recommended dose	Limiting amino acid
R1	R2	R3	R4	R5	AP	LA

**Screening 3**				
Others manufacture's intake recommendation	Allergens	Enzymes	Additives	B12 (micrograms)
OR	AG	EZY	ADD	B12

Serving	Degree of evidence (Australian Institute of Sport)	Certification	Frequency	Source of information
**SG**	DE	CT	FC	SI

✓: Complies with what is established in the legislation

WHEY

PR	CH	KCAL	PROT	AA	BCAA	HC	SUG	FAT	NA
**Whey isolate zero protein belgian chocolate flavor**	Life pro	415,5	76,55	L-Leucine 10,06<br>L-isoleucine 6,39<br>L-Valine 5,07<br>L-aspartic acid 10,71<br>L-glutamic acid 16,45<br>L-Serine 4,13<br>L-Glycine 1,69<br>L-histidine 1,60<br>L-arginine 1,97<br>L-threonine 6,58<br>L-Alanine 4,42<br>L-Proline 5,54<br>L-Tyrosine 2.82<br>L-methionine 2,16<br>L-cysteine 2,82<br>L-phenylalanine 2,82<br>L-Lysine 9,77<br>L-tryptophan 1,97	21,52	0,99	0,28 g	0,43	0,4

SG	DE	CT	FC	SI
30 g	A	No	1-2 serving per day	Web commercial house

Screening 1					Screening 2		Screening 3				
R1	R2	R3	R4	R5	AP	LA	OR	AG	EZY	ADD	B12
✓	✓	"Maintain or gain muscle mass" ✓	✓	No claims	22,965	No claims	No claims	Milk and soy	Cellulase<br>Amylase<br>Lactase<br>Lipase<br>Protease	Soy lecithin<br>Sucralose<br>Aromas	No claims

Abbreviations: Product(100gr)=PR; Comercial House=CH; Kilocalories=Kcal; Protein(g)=PROT; Aminogram(g)=AA; BCAA(g)=BCAA; Carbohydrates(g)=HC; Of which sugars(g)=SUG; Fats(g)=FAT; Sodium(mg)=NA
Screening 1: Regulation 1169/2011=R1; Royal Decree 1487/2009=R2; Regulation 432/2012=R3; World Anti-Doping Code=R5
Screening 2: Amount of protein recommended dose=AP; Limiting amino acid=LA
Screening 3: Others manufacture's intake recommendation=OR; Allergens=AG; Enzymes=EZY; Additives=ADD; B12 (μg)=B12
Use=USE; Serving=SG; Degree of evidence(Australian Institute of Sport)=DE; Certification=CT; Frequency=FC; Source of information=SI
✓Complies with what is established in the legislation.

PR	CH	KCAL	PROT	AA	BCAA	HC	SUG	FAT	NA
Whey Protein	Optimum nutrition	372	79	Valine, isoleucine, leucine (BCAAs: 5,5g), tryptophan, threonine lysine, phenylalanine, methionine: 11g Arginine, cysteine, tyrosine, histidine, proline, glutamine and glutamic acid: 7.7g Aspartic acid, serine, glycine, alanine: 5.3g	5,5	5,8	3,9 g	3,8	244

Screening 1					Screening 2		Screening 3				
R1	R2	R3	R4	R5	AP	LA	OR	AG	EZY	ADD	B12
✓	✓	"Development and care of muscle mass“ ✓	✓	No claims	23,7	No claims	No claims	Milk, soy and it is possible that it contains gluten, egg, nuts and peanut	Amylase Protease Cellulase Beta-D-Galactosidase Lipase	Soy lecithin sucralose acesulfame K, powder cocoa Aromas	No claims

SG	DE	CT	FC	SI
30 g	A	Yes	No claims	Label

Abbreviations: Product(100gr)=PR; Comercial House=CH; Kilocalories=Kcal; Protein(g)=PROT; Aminogram(g)=AA; BCAA(g)=BCAA; Carbohydrates(g)=HC; Of which sugars(g)=SUG; Fats(g)=FAT; Sodium(mg)=NA
Screening 1: Regulation 1169/2011=R1; Royal Decree 1487/2009=R2; Regulation 432/2012=R3; World Anti-Doping Code=R5
Screening 2: Amount of protein recommended dose=AP; Limiting amino acid=LA
Screening 3: Others manufacture's intake recommendation=OR; Allergens=AG; Enzymes=EZY; Additives=ADD; B12 (µg)=B12
Use=USE; Serving=SG; Degree of evidence(Australian Institute of Sport)=DE; Certification=CT; Frequency=FC; Source of information=SI
✓ Complies with what is established in the legislation.

PR	CH	KCAL	PROT	AA	BCAA	HC	SUG	FAT	NA
Whey isolate protein vanilla flavor (isolated and concentrated whey protein)	Decathlon	373	80	Alanine 3,9<br>Arginine 1,8<br>Aspartic acid 8,7<br>Glutamic acid 14,2<br>Cysteine 2<br>Glycine 1,3<br>Histidine 1,4<br>Isoleucine 5<br>Leucine 8.7<br>Lysine 7,4<br>Methionine 1,7<br>Phenylalanine 2,5<br>Proline 4,3<br>Serine 3.8<br>Threonine 5<br>Tryptophan 1,5<br>Tyrosine 2.4<br>Valine 4.5	18,2	6	5,8 g	3,2	440

SG	DE	CT	FC	SI
30 g	A	No	No claims	Label

Screening 1					Screening 2		Screening 3				
R1	R2	R3	R4	R5	AP	LA	OR	AG	EZY	ADD	B12
✓	✓	✓ "Development and care of muscle mass"	✓	No claims	24	No claims	No claims	Milk, soy and it possible that it contains gluten, egg, sulphites, celerey, walnuts and mustard	No claims	Soy and sunflower lecithin Skimmed milk powder Sucralose, aromas	No claims

Abbreviations: Product(100gr)=PR; Comercial House=CH; Kilocalories=Kcal; Protein(g)=PROT; Aminogram(g)=AA; BCAA(g)=BCAA; Carbohydrates(g)=HC; Of which sugars(g)=SUG; Fats(g)=FAT; Sodium(mg)=NA
Screening 1: Regulation 1169/2011=R1; Royal Decree 1487/2009=R2; Regulation 432/2012=R3; World Anti-Doping Code=R5
Screening 2: Amount of protein recommended dose=AP; Limiting amino acid=LA
Screening 3: Others manufacture's intake recommendation=OR; Allergens=AG; Enzymes=EZY; Additives=ADD; B12 (µg)=B12
Use=USE; Serving=SG; Degree of evidence(Australian Institute of Sport)=DE; Certification=CT; Frequency=FC; Source of information=SI
✓ Complies with what is established in the legislation.

PR	CH	KCAL	PROT	AA	BCAA	HC	SUG	FAT	NA
Whey protein chocolate flavor (native (whey protein isolate)	Decathlon	374	85	Alanine 4,2<br>Arginine 2,3<br>Aspartic acid 10<br>Glutamic acid 15,1<br>Cysteine 2,6<br>Glycine 1,7<br>Histidine 1,9<br>Isoleucine 4,7<br>Leucine 10.6<br>Lysine 8,5<br>Methionine 1,9<br>Phenylalanine 3,1<br>Proline 4,1<br>Serine 4.2<br>Threonine 4,4<br>Tryptophan 2<br>Tyrosine 3.2<br>Valine 4.5	19,8	4,8	0,5 g	1,7	164

SG	DE	CT	FC	SI
30 g	A	No	No claims	Label

Screening 1					Screening 2		Screening 3				
R1	R2	R3	R4	R5	AP	LA	OR	AG	EZY	ADD	B12
✓	✓	"Development and care of muscle mass" ✓	✓	No claims	25,5	No claims	No claims	Milk, soy and it possible that it contains gluten, egg, sulphites, celerey, walnuts and mustard	Lactase	Soy lecithin<br>Degreased cocoa powder<br>Aromas<br>Sucralose	No claims

Abbreviations: Product(100gr)=PR; Comercial House=CH; Kilocalories=Kcal; Protein(g)=PROT; Aminogram(g)=AA; BCAA(g)=BCAA; Carbohydrates(g)=HC; Of which sugars(g)=SUG; Fats(g)=FAT; Sodium(mg)=NA
Screening 1: Regulation 1169/2011=R1; Royal Decree 1487/2009=R2; Regulation 432/2012=R3; World Anti-Doping Code=R5
Screening 2: Amount of protein recommended dose=AP; Limiting amino acid=LA
Screening 3: Others manufacture's intake recommendation=OR; Allergens=AG; Enzymes=EZY; Additives=ADD; B12 (µg)=B12
Use=USE; Serving=SG; Degree of evidence(Australian Institute of Sport)=DE; Certification=CT; Frequency=FC; Source of information=SI
✓ Complies with what is established in the legislation.

PR	CH	KCAL	PROT	AA	BCAA	HC	SUG	FAT	NA
Whey protein chocolate flavor (whey protein isolate)	Corength Decathlon	376	81	No claims	No claims	11	1,4 g	0,9	192

SG	DE	CT	FC	SI
30 g	A	No	No claims	Label

Screening 1					Screening 2		Screening 3				
R1	R2	R3	R4	R5	AP	LA	OR	AG	EZY	ADD	B12
✓	✓	"Development and care of muscle mass" ✓	✓	No claims	24,3	No claims	No claims	Milk, soy and it possible that it contains gluten, egg, sulphites, celerey, walnuts and mustard	No claims	Sucralose L-carnitine Green tea Cocoa	No claims

Abbreviations: Product(100gr)=PR; Comercial House=CH; Kilocalories=Kcal; Protein(g)=PROT; Aminogram(g)=AA; BCAA(g)=BCAA; Carbohydrates(g)=HC; Of which sugars(g)=SUG; Fats(g)=FAT; Sodium(mg)=NA
Screening 1: Regulation 1169/2011=R1; Royal Decree 1487/2009=R2; Regulation 432/2012=R3; World Anti-Doping Code=R5
Screening 2: Amount of protein recommended dose=AP; Limiting amino acid=LA
Screening 3: Others manufacture's intake recommendation=OR; Allergens=AG; Enzymes=EZY; Additives=ADD; B12 (μg)=B12
Use=USE; Serving=SG; Degree of evidence(Australian Institute of Sport)=DE; Certification=CT; Frequency=FC; Source of information=SI
✓Complies with what is established in the legislation.

PR	CH	KCAL	PROT	AA	BCAA	HC	SUG	FAT	NA
Chocolate flavor protein (whey protein)	Aptonia	379	72	Alanine 3,6<br>Arginine 2,1<br>Aspartic acid 8,1<br>Glutamic acid 12,6<br>Cysteine 2,1<br>Glycine 1,4<br>Histidine 1,6<br>Isoleucine 4,1<br>Leucine 8.6<br>Lysine 7,2<br>Methionine 1,7<br>Phenylalanine 2,8<br>Proline 3.5<br>Serine 3.5<br>Threonine 3.9<br>Tryptophan 1,7<br>Tyrosine 2.7<br>Valine 4	16,7	6	Glucose syrup<br>3 g sugar	6,5	680

SG	DE	CT	FC	SI
21 g	A	No	3 times a day	Label

Screening 1					Screening 2		Screening 3				
R1	R2	R3	R4	R5	AP	LA	OR	AG	EZY	ADD	B12
No claims	No claims	No claims	No claims	No claims	15,12	No claims	No claims	No claims	No claims	Sunflower and soy lecithin (emulsifier)<br>Low-fat cocoa powder<br>Aromas<br>Silicon dioxide (anti-caking)<br>Stabilizers<br>Green tea extract<br>L-carnitine<br>Sucralose, potassium acelsulfame<br>Tocopherol (antioxidant)<br>Sodium caseinate<br>Tetrasodium diphosphate (stabilizer)<br>Konjac root extract<br>Carcamo oil	No claims

Abbreviations: Product(100gr)=PR; Comercial House=CH; Kilocalories=Kcal; Protein(g)=PROT; Aminogram(g)=AA; BCAA(g)=BCAA; Carbohydrates(g)=HC; Of which sugars(g)=SUG; Fats(g)=FAT; Sodium(mg)=NA
Screening 1: Regulation 1169/2011=R1; Royal Decree 1487/2009=R2; Regulation 432/2012=R3; World Anti-Doping Code=R5
Screening 2: Amount of protein recommended dose=AP; Limiting amino acid=LA
Screening 3: Others manufacture's intake recommendation=OR; Allergens=AG; Enzymes=EZY; Additives=ADD; B12 (µg)=B12
Use=USE; Serving=SG; Degree of evidence(Australian Institute of Sport)=DE; Certification=CT; Frequency=FC; Source of information=SI
✓Complies with what is established in the legislation.

PR	CH	KCAL	PROT	AA	BCAA	HC	SUG	FAT	NA
Raspberry flavor whey isolate protein (isolated and concentrated whey protein)	Decathlon	373	80	Alanine 3,9<br>Arginine 1,8<br>Aspartic acid 8,7<br>Glutamic acid 14,2<br>Cysteine 2<br>Glycine 1,3<br>Histidine 1,4<br>Isoleucine 5<br>Leucine 8.7<br>Lysine 7,4<br>Methionine 1,7<br>Phenylalanine 2,5<br>Proline 4,3<br>Serine 3.8<br>Threonine 5<br>Tryptophan 1,5<br>Tyrosine 2.4<br>Valine 4.5	18,2	6	5,8 g	3,2	440

SG	DE	CT	FC	SI
30 g	A	No	No claims	Label

Screening 1					Screening 2		Screening 3				
R1	R2	R3	R4	R5	AP	LA	OR	AG	EZY	ADD	B12
✓	✓	"Development and care of muscle mass" ✓	✓	No claims	24	No claims	No claims	Milk, soy and it possible that it contains gluten, egg, sulphites, celerey, walnuts and mustard	No claims	Soy and sunflower lecithin<br>Skimmed milk powder<br>Sucralose, aromas	No claims

Abbreviations: Product(100gr)=PR; Comercial House=CH; Kilocalories=Kcal; Protein(g)=PROT; Aminogram(g)=AA; BCAA(g)=BCAA; Carbohydrates(g)=HC; Of which sugars(g)=SUG; Fats(g)=FAT; Sodium(mg)=NA
Screening 1: Regulation 1169/2011=R1; Royal Decree 1487/2009=R2; Regulation 432/2012=R3; World Anti-Doping Code=R5
Screening 2: Amount of protein recommended dose=AP; Limiting amino acid=LA
Screening 3: Others manufacture's intake recommendation=OR; Allergens=AG; Enzymes=EZY; Additives=ADD; B12 (µg)=B12
Use=USE; Serving=SG; Degree of evidence(Australian Institute of Sport)=DE; Certification=CT; Frequency=FC; Source of information=SI
✓ Complies with what is established in the legislation.

PR	CH	KCAL	PROT	AA	BCAA	HC	SUG	FAT	NA
Whey isolate chocolate flavor protein (whey protein isolated and concentrated)	Decathlon	377	80	Alanine 3,9<br>Arginine 1,8<br>Aspartic acid 8,7<br>Glutamic acid 14,1<br>Cysteine 1,9<br>Glycine 1,3<br>Histidine 1,4<br>Isoleucine 5<br>Leucine 8.5<br>Lysine 7,5<br>Methionine 1,6<br>Phenylalanine 2,5<br>Proline 4,3<br>Serine 3.7<br>Threonine 5,2<br>Tryptophan 1,6<br>Tyrosine 2.4<br>Valine 4.5	18	5	3,3 g	3,8	440

SG	DE	CT	FC	SI
30 g	A	No	No claims	Label

Screening 1					Screening 2		Screening 3				
R1	R2	R3	R4	R5	AP	LA	OR	AG	EZY	ADD	B12
✓	✓	"Development and care of muscle mass" ✓	✓	No claims	24	No claims	No claims	Milk, soy and it possible that it contains gluten, egg, sulphites, celerey, walnuts and mustard	No claims	Soy and sunflower lecithin<br>Skimmed milk powder<br>Sucralose, aromas<br>Degreased cocoa powder	No claims

Abbreviations: Product(100gr)=PR; Comercial House=CH; Kilocalories=Kcal; Protein(g)=PROT; Aminogram(g)=AA; BCAA(g)=BCAA; Carbohydrates(g)=HC; Of which sugars(g)=SUG; Fats(g)=FAT; Sodium(mg)=NA
Screening 1: Regulation 1169/2011=R1; Royal Decree 1487/2009=R2; Regulation 432/2012=R3; World Anti-Doping Code=R5
Screening 2: Amount of protein recommended dose=AP; Limiting amino acid=LA
Screening 3: Others manufacture's intake recommendation=OR; Allergens=AG; Enzymes=EZY; Additives=ADD; B12 (µg)=B12
Use=USE; Serving=SG; Degree of evidence(Australian Institute of Sport)=DE; Certification=CT; Frequency=FC; Source of information=SI
✓ Complies with what is established in the legislation.

PR	CH	KCAL	PROT	AA	BCAA	HC	SUG	FAT	NA
Whey isolate protein flavor cookie and cream	Decathlon	377	80	Alanine 3,9<br>Arginine 1,8<br>Aspartic acid 8,7<br>Glutamic acid 14,1<br>Cysteine 1,9<br>Glycine 1,3<br>Histidine 1,4<br>Isoleucine 5<br>Leucine 8.5<br>Lysine 7,5<br>Methionine 1,6<br>Phenylalanine 2,5<br>Proline 4,3<br>Serine 3.7<br>Threonine 5,2<br>Tryptophan 1,6<br>Tyrosine 2.4<br>Valine 4.5	18	5	3,3 g	3,8	440

SG	DE	CT	FC	SI
30 g	A	No	No claims	Label

Screening 1					Screening 2		Screening 3				
R1	R2	R3	R4	R5	AP	LA	OR	AG	EZY	ADD	B12
✓	✓	"Development and care of muscle mass" ✓	✓	No claims	24	No claims	No claims	Milk, soy and it possible that it contains gluten, egg, sulphites, celerey, walnuts and mustard	No claims	Soy and sunflower lecithin<br>Skimmed milk powder<br>Sucralose, aromas<br>Degreased cocoa powder	No claims

Abbreviations: Product(100gr)=PR; Comercial House=CH; Kilocalories=Kcal; Protein(g)=PROT; Aminogram(g)=AA; BCAA(g)=BCAA; Carbohydrates(g)=HC; Of which sugars(g)=SUG; Fats(g)=FAT; Sodium(mg)=NA
Screening 1: Regulation 1169/2011=R1; Royal Decree 1487/2009=R2; Regulation 432/2012=R3; World Anti-Doping Code=R5
Screening 2: Amount of protein recommended dose=AP; Limiting amino acid=LA
Screening 3: Others manufacture's intake recommendation=OR; Allergens=AG; Enzymes=EZY; Additives=ADD; B12 (µg)=B12
Use=USE; Serving=SG; Degree of evidence(Australian Institute of Sport)=DE; Certification=CT; Frequency=FC; Source of information=SI
✓Complies with what is established in the legislation.

PR	CH	KCAL	PROT	AA	BCAA	HC	SUG	FAT	NA
Whey isolate vanilla flavor protein (concentrated whey protein)	Decathlon	388	69	Alanine 3,4<br>Arginine 1,8<br>Aspartic acid 8<br>Glutamic acid 12<br>Cysteine 3,1<br>Glycine 1,2<br>Histidine 1,4<br>Isoleucine 3.9<br>Leucine 7.7<br>Lysine 6,4<br>Phenylalanine 4,3<br>Proline 3.5<br>Serine 3.3<br>Threonine 4,2<br>Tryptophan 1,3<br>Tyrosine 3.7<br>Valine 4.5	16,1	15	5,6	5,3	440

SG	DE	CT	FC	SI
30 g	A	No	No claims	Label

Screening 1					Screening 2		Screening 3				
R1	R2	R3	R4	R5	AP	LA	OR	AG	EZY	ADD	B12
✓	✓	"Development and care of muscle mass" ✓	✓	No claims	20,7	No claims	No claims	Milk, soy and it possible that it contains gluten, egg, sulphites, celerey, walnuts and mustard	No claims	Soy lecithin<br>Maltodextrin<br>Sucralose<br>Acesulfame Potassium<br>Xanthan Gum<br>Aromas	No claims

Abbreviations: Product(100gr)=PR; Comercial House=CH; Kilocalories=Kcal; Protein(g)=PROT; Aminogram(g)=AA; BCAA(g)=BCAA; Carbohydrates(g)=HC; Of which sugars(g)=SUG; Fats(g)=FAT; Sodium(mg)=NA
Screening 1: Regulation 1169/2011=R1; Royal Decree 1487/2009=R2; Regulation 432/2012=R3; World Anti-Doping Code=R5
Screening 2: Amount of protein recommended dose=AP; Limiting amino acid=LA
Screening 3: Others manufacture's intake recommendation=OR; Allergens=AG; Enzymes=EZY; Additives=ADD; B12 (μg)=B12
Use=USE; Serving=SG; Degree of evidence(Australian Institute of Sport)=DE; Certification=CT; Frequency=FC; Source of information=SI
✓ Complies with what is established in the legislation.

PR	CH	KCAL	PROT	AA	BCAA	HC	SUG	FAT	NA
Chocolate flavored whey protein (concentrated whey protein)	Decathlon	394	70	Alanine 3,4<br>Arginine 1,8<br>Aspartic acid 7,9<br>Glutamic acid 12,2<br>Methionine 2,8<br>Glycine 1,3<br>Histidine 1,4<br>Isoleucine 4<br>Leucine 7.9<br>Lysine 6.7<br>Phenylalanine 4,5<br>Proline 3.8<br>Serine 3.3<br>Threonine 4,1<br>Tryptophan 1,3<br>Valine 3.8	15,7	15	5 g	5,5	60

Screening 1					Screening 2		Screening 3				
R1	R2	R3	R4	R5	AP	LA	OR	AG	EZY	ADD	B12
✓	✓	"Development and care of muscle mass ✓	✓	No claims	70	No claims		Milk, soy and it possible that it contains gluten, egg, sulphites, celerey, walnuts and mustard	No claims	Sunflower lecithin<br>Maltodextrin<br>Degreased cocoa powder<br>Aromas<br>Xanthan gum<br>Salt<br>Acesulfame potassium<br>Sucralose	No claims

SG	DE	CT	FC	SI
30 g	A	No	No claims	Label

Abbreviations: Product(100gr)=PR; Comercial House=CH; Kilocalories=Kcal; Protein(g)=PROT; Aminogram(g)=AA; BCAA(g)=BCAA; Carbohydrates(g)=HC; Of which sugars(g)=SUG; Fats(g)=FAT; Sodium(mg)=NA
Screening 1: Regulation 1169/2011=R1; Royal Decree 1487/2009=R2; Regulation 432/2012=R3; World Anti-Doping Code=R5
Screening 2: Amount of protein recommended dose=AP; Limiting amino acid=LA
Screening 3: Others manufacture's intake recommendation=OR; Allergens=AG; Enzymes=EZY; Additives=ADD; B12 (µg)=B12
Use=USE; Serving=SG; Degree of evidence(Australian Institute of Sport)=DE; Certification=CT; Frequency=FC; Source of information=SI
✓ Complies with what is established in the legislation.

PR	CH	KCAL	PROT	AA	BCAA	HC	SUG	FAT	NA
Chocolate flavored whey protein (whey protein)	Isostar	387	72	No claims	No claims	7,6	2,4 g	6,6	600

*Does not comply with the Regulation.

SG	DE	CT	FC	SI
30 g	A	No	No claims	Label

Screening 1					Screening 2		Screening 3				
R1	R2	R3	R4	R5	AP	LA	OR	AG	EZY	ADD	B12
✓	✓	"Improves physical performance when training" *	No claims	No claims	21,6	No claims	No claims	Milk, wheat, egg, soy, lactose, whey	No claims	Sunflower and soy lecithin<br>Degreased cocoa powder<br>Aroma (coloring E150c)<br>Calcium phosphate<br>Aspartame<br>Acesulfame K	No claims

Abbreviations: Product(100gr)=PR; Comercial House=CH; Kilocalories=Kcal; Protein(g)=PROT; Aminogram(g)=AA; BCAA(g)=BCAA; Carbohydrates(g)=HC; Of which sugars(g)=SUG; Fats(g)=FAT; Sodium(mg)=NA
Screening 1: Regulation 1169/2011=R1; Royal Decree 1487/2009=R2; Regulation 432/2012=R3; World Anti-Doping Code=R5
Screening 2: Amount of protein recommended dose=AP; Limiting amino acid=LA
Screening 3: Others manufacture's intake recommendation=OR; Allergens=AG; Enzymes=EZY; Additives=ADD; B12 (µg)=B12
Use=USE; Serving=SG; Degree of evidence(Australian Institute of Sport)=DE; Certification=CT; Frequency=FC; Source of information=SI
✓ Complies with what is established in the legislation.

PR	CH	KCAL	PROT	AA	BCAA	HC	SUG	FAT	NA
Whey concentrate and isolate (whey protein) chocolate flavor	Weider	385	76	Alanine 4 Arginine 2,1 Aspartic acid 8,7 Cysteine 1,9 L-glutamine 24,3 Glycine 1,5 Histidine 1,4 L-Isoleucine 5,1 L-Leucine 8,6 L-Lysine 7,6 L-Methionine 1,7 L-Phenylalanine 2,6 L-Proline 5,3 L-Serine 4 L-Threonine 5,8 L-Tryptophan 1,4 L-Tyrosine 2,4 L-Valine 4,7	18,4	6	4,8 g	6,3	220

SG	DE	CT	FC	SI
30 g	A	No	No claims	Label

Screening 1					Screening 2		Screening 3				
R1	R2	R3	R4	R5	AP	LA	OR	AG	EZY	ADD	B12
✓	✓	"Development and care of muscle mass" ✓	✓	No claims	22,8	No claims	No claims	Milk	Amylase Protease Cellulase Lipase Lactase	Sunflower lecithin Cocoa seeds powder Aromas Carboxymethylcellulose sodium Sucralose Steviol glycosides Cocoa extract	No claims

Abbreviations: Product(100gr)=PR; Comercial House=CH; Kilocalories=Kcal; Protein(g)=PROT; Aminogram(g)=AA; BCAA(g)=BCAA; Carbohydrates(g)=HC; Of which sugars(g)=SUG; Fats(g)=FAT; Sodium(mg)=NA
Screening 1: Regulation 1169/2011=R1; Royal Decree 1487/2009=R2; Regulation 432/2012=R3; World Anti-Doping Code=R5
Screening 2: Amount of protein recommended dose=AP; Limiting amino acid=LA
Screening 3: Others manufacture's intake recommendation=OR; Allergens=AG; Enzymes=EZY; Additives=ADD; B12 (µg)=B12
Use=USE; Serving=SG; Degree of evidence(Australian Institute of Sport)=DE; Certification=CT; Frequency=FC; Source of information=SI
✓ Complies with what is established in the legislation.

PR	CH	KCAL	PROT	AA	BCAA	HC	SUG	FAT	NA
Whey isolate protein zero white chocolate flavor	Life pro	371,4	87,9	L-Leucine 10,06<br>L-isoleucine 6,39<br>L-Valine 5,07<br>L-aspartic acid 10,71<br>L-glutamic acid 16,45<br>L-Serine 4,13<br>L-Glycine 1,69<br>L-histidine 1,60<br>L-arginine 1,97<br>L-threonine 6,58<br>L-Alanine 4,42<br>L-Proline 5,54<br>L-Tyrosine 2.82<br>L-methionine 2,16<br>L-cysteine 2,82<br>L-phenylalanine 2,82<br>L-Lysine 9,77<br>L-tryptophan 1,97	20,15	4,3	0,8 g	0,88	No claims

SG	DE	CT	FC	SI
30 g	A	No	1-2 times a day	Web commercial house

Screening 1					Screening 2		Screening 3				
R1	R2	R3	R4	R5	AP	LA	OR	AG	EZY	ADD	B12
✓	✓	"Maintain or gain muscle mass" ✓	✓	No claims	26,37	No claims	No claims	Whey, soy	Amylase<br>Cellulase<br>Lactase<br>Lipase<br>Protease	Soy lecithin<br>Aromas<br>Sucralose	No claims

Abbreviations: Product(100gr)=PR; Comercial House=CH; Kilocalories=Kcal; Protein(g)=PROT; Aminogram(g)=AA; BCAA(g)=BCAA; Carbohydrates(g)=HC; Of which sugars(g)=SUG; Fats(g)=FAT; Sodium(mg)=NA
Screening 1: Regulation 1169/2011=R1; Royal Decree 1487/2009=R2; Regulation 432/2012=R3; World Anti-Doping Code=R5
Screening 2: Amount of protein recommended dose=AP; Limiting amino acid=LA
Screening 3: Others manufacture's intake recommendation=OR; Allergens=AG; Enzymes=EZY; Additives=ADD; B12 (µg)=B12
Use=USE; Serving=SG; Degree of evidence(Australian Institute of Sport)=DE; Certification=CT; Frequency=FC; Source of information=SI
✓ Complies with what is established in the legislation.

PR	CH	KCAL	PROT	AA	BCAA	HC	SUG	FAT	NA
Whey protein	Life pro	333,45	72,37	L-Leucine 8,41<br>L-Isoleucine 4,87<br>L-Valine 4.87<br>L-Aspartic Acid 8,34<br>L-Glutamic Acid 13,58<br>L-Serine 4,2<br>L-Glycine 1,47<br>L-Histidine 1,40<br>L-arginine 1,99<br>L-threonine 5,53<br>L-Alanine 4.05<br>L-Proline 4.87<br>L-Tyrosine 2,36<br>L-Methionine 1,69<br>L-Cysteine 1,77<br>L-Phenylalanine 2,52 L-Lysine 7,30<br>L-Tryptophan 1,40	18,15	7,04	2,66 g	4,21	No claims

Screening 1					Screening 2		Screening 3				
R1	R2	R3	R4	R5	AP	LA	OR	AG	EZY	ADD	B12
✓	✓	"Maintain or gain muscle mass" ✓	✓	No claims	21,711	No claims	Before or after training or at another time of day; on diets to lose weight or increase muscle mass	Whey, soy	Amylase<br>Cellulase<br>Lactase<br>Lipase<br>Protease	Soy lecithin<br>Aromas<br>Sucralose<br>Degreased Cocoa Powder Instant<br>Egg White Powder<br>Xanthan Gum Salt	No claims

SG	DE	CT	FC	SI
30 g	A	No	1-2 times a day	Web commercial house

Abbreviations: Product(100gr)=PR; Comercial House=CH; Kilocalories=Kcal; Protein(g)=PROT; Aminogram(g)=AA; BCAA(g)=BCAA; Carbohydrates(g)=HC; Of which sugars(g)=SUG; Fats(g)=FAT; Sodium(mg)=NA
Screening 1: Regulation 1169/2011=R1; Royal Decree 1487/2009=R2; Regulation 432/2012=R3; World Anti-Doping Code=R5
Screening 2: Amount of protein recommended dose=AP; Limiting amino acid=LA
Screening 3: Others manufacture's intake recommendation=OR; Allergens=AG; Enzymes=EZY; Additives=ADD; B12 (μg)=B12
Use=USE; Serving=SG; Degree of evidence(Australian Institute of Sport)=DE; Certification=CT; Frequency=FC; Source of information=SI
✓ Complies with what is established in the legislation.

PR	CH	KCAL	PROT	AA	BCAA	HC	SUG	FAT	NA
Hydrolyzed whey protein (isolate) flavor cookies and cream	Sascha fitness	314	71,42	Leucine 7,23<br>L-isoleucine 4,2<br>L-valine 3,92<br>L-alanine 3,26<br>L-aspartic acid 7,54<br>L-glutamine 11,84<br>L-tyrosine 2,08<br>L-arginine 1,77<br>L-cysteine 1,42<br>L-glycine 1,488<br>L-lysine 6,237<br>L-phenylalanine 2,32<br>L-serine 3,81<br>L-tryptophan 1,51<br>L-histidine 1,36<br>L-methionine 1,63<br>L-proline 5,068<br>L-threonine 4,7	15,35	1	No claims	No claims	271

SG	DE	CT	FC	SI
31,3 g	A	No	1-2 times a day	Web commercial house

Screening 1					Screening 2		Screening 3				
R1	R2	R3	R4	R5	AP	LA	OR	AG	EZY	ADD	B12
✓	✓	"Maintain or gain muscle mass" ✓	✓	No claims	22,35	No claims	No claims	No claims	Protease<br>Lipase<br>Amylase<br>Lactase	Cocoa<br>Stevia Extract<br>Xanthan gum<br>Sunflower lecithin<br>N&A flavor	No claims

Abbreviations: Product(100gr)=PR; Comercial House=CH; Kilocalories=Kcal; Protein(g)=PROT; Aminogram(g)=AA; BCAA(g)=BCAA; Carbohydrates(g)=HC; Of which sugars(g)=SUG; Fats(g)=FAT; Sodium(mg)=NA
Screening 1: Regulation 1169/2011=R1; Royal Decree 1487/2009=R2; Regulation 432/2012=R3; World Anti-Doping Code=R5
Screening 2: Amount of protein recommended dose=AP; Limiting amino acid=LA
Screening 3: Others manufacture's intake recommendation=OR; Allergens=AG; Enzymes=EZY; Additives=ADD; B12 (µg)=B12
Use=USE; Serving=SG; Degree of evidence(Australian Institute of Sport)=DE; Certification=CT; Frequency=FC; Source of information=SI
✓ Complies with what is established in the legislation.

PR	CH	KCAL	PROT	AA	BCAA	HC	SUG	FAT	NA
Whey isolate protein	My protein	359	81	No claims	No claims	4,63 g		1,1	200

*Does not comply with the Regulation.

SG	DE	CT	FC	SI
25 g	A	Si	No claims	Web commercial house

Screening 1					Screening 2		Screening 3				
R1	R2	R3	R4	R5	AP	LA	OR	AG	EZY	ADD	B12
✓	✓	"Protein: maintenance and increase of muscle mass" Creatine: "improves performance" *	No claims	No claims	20,25	No claims	Minutes after training or at any time of the day	Milk, soy, barley (in Banoffee flavor; chocolate and caramel; latte), sulphites (blueberry flavor; blueberry and raspberry flavor), wheat (birthday cake flavor)	No claims	Soy lecithin Flavoring Sucralose	No claims

Abbreviations: Product(100gr)=PR; Comercial House=CH; Kilocalories=Kcal; Protein(g)=PROT; Aminogram(g)=AA; BCAA(g)=BCAA; Carbohydrates(g)=HC; Of which sugars(g)=SUG; Fats(g)=FAT; Sodium(mg)=NA
Screening 1: Regulation 1169/2011=R1; Royal Decree 1487/2009=R2; Regulation 432/2012=R3; World Anti-Doping Code=R5
Screening 2: Amount of protein recommended dose=AP; Limiting amino acid=LA
Screening 3: Others manufacture's intake recommendation=OR; Allergens=AG; Enzymes=EZY; Additives=ADD; B12 (µg)=B12
Use=USE; Serving=SG; Degree of evidence(Australian Institute of Sport)=DE; Certification=CT; Frequency=FC; Source of information=SI
✓Complies with what is established in the legislation.

PR	CH	KCAL	PROT	AA	BCAA	HC	SUG	FAT	NA
Chocolate and coconut flavored whey protein (whey protein isolate and concentrate)	Foodspring	381	76,2	Alanine 3,802<br>Arginine 1,996<br>Aspartic acid 8,092<br>Cysteine 2,278<br>Glutamic acid 12, 565<br>Glycine 1,372<br>Histidine 1,372<br>Isoleucine 4,633<br>Leucine 8,595<br>Lysine 6,934<br>Methionine 1,737<br>Phenylalanine 2,484<br>Proline 3,962<br>Serine 3,490<br>Threonine 4,557<br>Tryptophan 1,524<br>Tyrosine 2,591<br>Valine 4,206	17,434	5,4	4,2 g	5,4	264

SG	DE	CT	FC	SI
30 g	A	No	1-3 times a day	Web commercial house

Screening 1					Screening 2		Screening 3				
R1	R2	R3	R4	R5	AP	LA	OR	AG	EZY	ADD	B12
✓	✓	"Support for muscle development" ✓	✓	No claims	22,86	No claims	Useful if you train strength, endurance or want to gain muscle mass	Whey, lactose	No claims	Sucralose Steviol Glycoside<br>Sunflower Lecithin<br>Cocoa Powder<br>Aromas<br>Coconut flakes	No claims

Abbreviations: Product(100gr)=PR; Comercial House=CH; Kilocalories=Kcal; Protein(g)=PROT; Aminogram(g)=AA; BCAA(g)=BCAA; Carbohydrates(g)=HC; Of which sugars(g)=SUG; Fats(g)=FAT; Sodium(mg)=NA
Screening 1: Regulation 1169/2011=R1; Royal Decree 1487/2009=R2; Regulation 432/2012=R3; World Anti-Doping Code=R5
Screening 2: Amount of protein recommended dose=AP; Limiting amino acid=LA
Screening 3: Others manufacture's intake recommendation=OR; Allergens=AG; Enzymes=EZY; Additives=ADD; B12 (µg)=B12
Use=USE; Serving=SG; Degree of evidence(Australian Institute of Sport)=DE; Certification=CT; Frequency=FC; Source of information=SI
✓ Complies with what is established in the legislation.

PR	CH	KCAL	PROT	AA	BCAA	HC	SUG	FAT	NA
Whey Protein Concentrate Double Chocolate Flavor	FullGas	367	66	Aspartic acid 9,14<br>Glutamic acid 15,42<br>Alanine 6,39<br>Arginine 4,22<br>Cysteine 1,38<br>Phenylalanine 2,9<br>Glycine 9.04<br>Histidine 1,38<br>Isoleucine 4.73<br>Leucine 8.02<br>Lysine 7,47<br>Methionine 1,6<br>Proline 8.55<br>Serine 4.41<br>Tyrosine 2.04<br>Threonine 5,19<br>Tryptophan 1,29<br>Valine 4,81<br>Hydroxylysine 0,39<br>Hydroxyproline 4,21	17,56	5,9	3,5 g	7,7	216

*Does not comply with the Regulation.

SG	DE	CT	FC	SI
40 g	A	No	No claims	Web commercial house

Screening 1					Screening 2		Screening 3				
R1	R2	R3	R4	R5	AP	LA	OR	AG	EZY	ADD	B12
✓	✓	"Muscle mass growth and to recovery" *	No claims	No claims	26,4	No claims	Useful for gaining muscle mass and in recovery	Milk, soy	No claims	Degreased cocoa powder<br>Aroma<br>Sucralose	No claims

Abbreviations: Product(100gr)=PR; Comercial House=CH; Kilocalories=Kcal; Protein(g)=PROT; Aminogram(g)=AA; BCAA(g)=BCAA; Carbohydrates(g)=HC; Of which sugars(g)=SUG; Fats(g)=FAT; Sodium(mg)=NA
Screening 1: Regulation 1169/2011=R1; Royal Decree 1487/2009=R2; Regulation 432/2012=R3; World Anti-Doping Code=R5
Screening 2: Amount of protein recommended dose=AP; Limiting amino acid=LA
Screening 3: Others manufacture's intake recommendation=OR; Allergens=AG; Enzymes=EZY; Additives=ADD; B12 (µg)=B12
Use=USE; Serving=SG; Degree of evidence(Australian Institute of Sport)=DE; Certification=CT; Frequency=FC; Source of information=SI
✓ Complies with what is established in the legislation.

PR	CH	KCAL	PROT	AA	BCAA	HC	SUG	FAT	NA
Neutral isopep optipep (hydrolyzed whey protein isolate)	FullGas	372	89	Aspartic acid 11,99<br>Glutamic acid 20,48<br>Alanine 5,94<br>Arginine 2,14<br>Cysteine 2,68<br>Phenylalanine 3,17<br>Glycine 1,71<br>Histidine 1,74<br>Isoleucine 8,21<br>Leucine 11.17<br>Lysine 11.03<br>Methionine 2,43<br>Proline 6,83<br>Serine 5.61<br>Tyrosine 3.02<br>Threonine 8<br>Tryptophan 1,81<br>Valine 7,49	26,87	1	1 g	1,5	172

*Does not comply with the Regulation.

SG	DE	CT	FC	SI
40 g	A	No		Web commercial house

Screening 1					Screening 2		Screening 3				
R1	R2	R3	R4	R5	AP	LA	OR	AG	EZY	ADD	B12
✓	✓	"Avoid breakdown of muscle tissue" *	No claims	No claims	35,6	No claims	Before or during training	Milk, hydrolyzed whey, soy	No claims	Soy lecithin	No claims

Abbreviations: Product(100gr)=PR; Comercial House=CH; Kilocalories=Kcal; Protein(g)=PROT; Aminogram(g)=AA; BCAA(g)=BCAA; Carbohydrates(g)=HC; Of which sugars(g)=SUG; Fats(g)=FAT; Sodium(mg)=NA
Screening 1: Regulation 1169/2011=R1; Royal Decree 1487/2009=R2; Regulation 432/2012=R3; World Anti-Doping Code=R5
Screening 2: Amount of protein recommended dose=AP; Limiting amino acid=LA
Screening 3: Others manufacture's intake recommendation=OR; Allergens=AG; Enzymes=EZY; Additives=ADD; B12 (μg)=B12
Use=USE; Serving=SG; Degree of evidence(Australian Institute of Sport)=DE; Certification=CT; Frequency=FC; Source of information=SI
✓ Complies with what is established in the legislation.

PR	CH	KCAL	PROT	AA	BCAA	HC	SUG	FAT	NA
Hydro 22 (hydrolyzed whey protein concentrate)	FullGas	396	74	Aspartic acid 11,02<br>Glutamic acid 19,12<br>Alanine 5,67<br>Arginine 2,64<br>Cysteine 2,36<br>Phenylalanine 3,50<br>Glycine 1,94<br>Histidine 1,84<br>Isoleucine 6.56<br>Leucine 11.61<br>Lysine 10,2<br>Methionine 1,99<br>Proline 6,42<br>Serine 5.46<br>Tyrosine 3,12<br>Threonine 7,52<br>Tryptophan 1.02<br>Valine 6,41	24,58	No claims	No claims	No claims	No claims

Screening 1					Screening 2		Screening 3				
R1	R2	R3	R4	R5	AP	LA	OR	AG	EZY	ADD	B12
✓	✓	No claims	No claims	No claims	29,6	No claims	No claims	Milk	No claims	No claims	No claims

SG	DE	CT	FC	SI
40 g	A	No	No claims	Web commercial house

Abbreviations: Product(100gr)=PR; Comercial House=CH; Kilocalories=Kcal; Protein(g)=PROT; Aminogram(g)=AA; BCAA(g)=BCAA; Carbohydrates(g)=HC; Of which sugars(g)=SUG; Fats(g)=FAT; Sodium(mg)=NA
Screening 1: Regulation 1169/2011=R1; Royal Decree 1487/2009=R2; Regulation 432/2012=R3; World Anti-Doping Code=R5
Screening 2: Amount of protein recommended dose=AP; Limiting amino acid=LA
Screening 3: Others manufacture's intake recommendation=OR; Allergens=AG; Enzymes=EZY; Additives=ADD; B12 (µg)=B12
Use=USE; Serving=SG; Degree of evidence(Australian Institute of Sport)=DE; Certification=CT; Frequency=FC; Source of information=SI
✓ Complies with what is established in the legislation.

PR	CH	KCAL	PROT	AA	BCAA	HC	SUG	FAT	NA
Whey Protein Isolate Double Chocolate Flavor	FullGas	346	74	Aspartic acid 11<br>Glutamic acid 18,1<br>Alanine 5<br>Arginine 2,1<br>Cysteine 2,2<br>Phenylalanine 3<br>Glycine 1,4<br>Histidine 1,4<br>Isoleucine 6,4<br>Leucine 10.6<br>Lysine 9,6<br>Methionine 2,2<br>Proline 5.5<br>Serine 4.6<br>Tyrosine 2.6<br>Threonine 6.7<br>Tryptophan 1,4<br>Valine 5.9	22,9	4,7	2,3 g	2,3	256

*Does not comply with the Regulation.

SG	DE	CT	FC	SI
40 g	A	No	No claims	Web commercial house

Screening 1					Screening 2		Screening 3				
R1	R2	R3	R4	R5	AP	LA	OR	AG	EZY	ADD	B12
✓	✓	"Muscle growth and recovery" *	No claims	No claims	29,6	No claims	Helpful in muscle mass gain and recovery	Milk, soy	No claims	Degreased cocoa powder<br>Aroma<br>Sucralose	No claims

Abbreviations: Product(100gr)=PR; Comercial House=CH; Kilocalories=Kcal; Protein(g)=PROT; Aminogram(g)=AA; BCAA(g)=BCAA; Carbohydrates(g)=HC; Of which sugars(g)=SUG; Fats(g)=FAT; Sodium(mg)=NA
Screening 1: Regulation 1169/2011=R1; Royal Decree 1487/2009=R2; Regulation 432/2012=R3; World Anti-Doping Code=R5
Screening 2: Amount of protein recommended dose=AP; Limiting amino acid=LA
Screening 3: Others manufacture's intake recommendation=OR; Allergens=AG; Enzymes=EZY; Additives=ADD; B12 (μg)=B12
Use=USE; Serving=SG; Degree of evidence(Australian Institute of Sport)=DE; Certification=CT; Frequency=FC; Source of information=SI
✓ Complies with what is established in the legislation.

PR	CH	KCAL	PROT	AA	BCAA	HC	SUG	FAT	NA
WPC/WPI (concentrado de proteína/aislado de proteínas) sabor vainilla	Performance sport nutrition	399	79	Alanine 3.9 Arginine 1.6 Aspartic acid 8.6 Cysteine 1.7 Glutamic acid 14.2 Glycine 1.1 Histidine 1.3 Isoleucine 5 Leucine 8.3 Lysine 7.5 Methionine 1.7 Phenylalanine 2.4 Proline 4.3 Serine 3.6 Threonine 5.3 Tryptophan 1.1 Tyrosine 2 Valine 4.6	17,8	5,5	3,8 g azúcar	7	320

SG	DE	CT	FC	SI
30,7 g	A	No	No claims	label

Screening 1					Screening 2		Screening 3				
R1	R2	R3	R4	R5	AP	LA	OR	AG	EZY	ADD	B12
✓	✓	"Recovery and muscle maintenance" NO OK	No claims	No claims	24,253	No claims	No claims	Milk, soya	No claims	Soy lecithin Sucralose Sodium chloride	No claims

Abbreviations: Product(100gr)=PR; Comercial House=CH; Kilocalories=Kcal; Protein(g)=PROT; Aminogram(g)=AA; BCAA(g)=BCAA; Carbohydrates(g)=HC; Of which sugars(g)=SUG; Fats(g)=FAT; Sodium(mg)=NA
Screening 1: Regulation 1169/2011=R1; Royal Decree 1487/2009=R2; Regulation 432/2012=R3; World Anti-Doping Code=R5
Screening 2: Amount of protein recommended dose=AP; Limiting amino acid=LA
Screening 3: Others manufacture's intake recommendation=OR; Allergens=AG; Enzymes=EZY; Additives=ADD; B12 (µg)=B12
Use=USE; Serving=SG; Degree of evidence(Australian Institute of Sport)=DE; Certification=CT; Frequency=FC; Source of information=SI
✓ Complies with what is established in the legislation.

PR	CH	KCAL	PROT	AA	BCAA	HC	SUG	FAT	NA
WPC/WPI (concentrado de proteína/aislado de proteínas) sabor vainilla	Performance sport nutrition	399	79	Alanine 3.9<br>Arginine 1.6<br>Aspartic acid 8.6<br>Cysteine 1.7<br>Glutamic acid 14.2<br>Glycine 1.1<br>Histidine 1.3<br>Isoleucine 5<br>Leucine 8.3<br>Lysine 7.5<br>Methionine 1.7<br>Phenylalanine 2.4<br>Proline 4.3<br>Serine 3.6<br>Threonine 5.3<br>Tryptophan 1.1<br>Tyrosine 2<br>Valine 4.6	17,8	5,5	3,8 g azúcar	7	320

*Does not comply with the Regulation.

SG	DE	CT	FC	SI
30,7 g	A	No	No claims	Label

Screening 1					Screening 2		Screening 3				
R1	R2	R3	R4	R5	AP	LA	OR	AG	EZY	ADD	B12
✓	✓	"Recovery and muscle maintenance" *	No claims	No claims	24,25<br>3	No claims	No claims	Milk, soya	No claims	Soy lecithin<br>Sucralose<br>Sodium chloride	No claims

Abbreviations: Product(100gr)=PR; Comercial House=CH; Kilocalories=Kcal; Protein(g)=PROT; Aminogram(g)=AA; BCAA(g)=BCAA; Carbohydrates(g)=HC; Of which sugars(g)=SUG; Fats(g)=FAT; Sodium(mg)=NA
Screening 1: Regulation 1169/2011=R1; Royal Decree 1487/2009=R2; Regulation 432/2012=R3; World Anti-Doping Code=R5
Screening 2: Amount of protein recommended dose=AP; Limiting amino acid=LA
Screening 3: Others manufacture's intake recommendation=OR; Allergens=AG; Enzymes=EZY; Additives=ADD; B12 (µg)=B12
Use=USE; Serving=SG; Degree of evidence(Australian Institute of Sport)=DE; Certification=CT; Frequency=FC; Source of information=SI
✓ Complies with what is established in the legislation.

PR	CH	KCAL	PROT	AA	BCAA	HC	SUG	FAT	NA
WPI (protein isolate) lemon flavor	Hero tech nutrition	375,38	86,96	Alanine 5.07<br>Arginine 2,2<br>Aspartic acid 10,96<br>Cysteine 2,49<br>Glutamic acid 18,55<br>Glycine 1,54<br>Histidine 1,69<br>Isoleucine 6,35<br>Leucine 10.71<br>Lysine 10,12<br>Methionine 2,19<br>Phenylalanine 3,1<br>Proline 5.77<br>Serine 4.42<br>Threonine 6.76<br>Tryptophan 1,55<br>Tyrosine 2.77<br>Valine 3.74	20,8	3,32	1,93 g	1,13	628

SG	DE	CT	FC	SI
30 g	A	No	No claims	Label

Screening 1					Screening 2		Screening 3				
R1	R2	R3	R4	R5	AP	LA	OR	AG	EZY	ADD	B12
✓	✓	"It contributes to the maintenance of bones in normal conditions" ✓	✓	No claims	26	No claims	No claims	Milk, soy	No claims	Soy lecithin<br>Aromas<br>Lemon juice powder<br>Sodium chloride<br>Citric acid<br>Coloring: curcumin<br>Sucralose<br>Acesulfame K	No claims

Abbreviations: Product(100gr)=PR; Comercial House=CH; Kilocalories=Kcal; Protein(g)=PROT; Aminogram(g)=AA; BCAA(g)=BCAA; Carbohydrates(g)=HC; Of which sugars(g)=SUG; Fats(g)=FAT; Sodium(mg)=NA
Screening 1: Regulation 1169/2011=R1; Royal Decree 1487/2009=R2; Regulation 432/2012=R3; World Anti-Doping Code=R5
Screening 2: Amount of protein recommended dose=AP; Limiting amino acid=LA
Screening 3: Others manufacture's intake recommendation=OR; Allergens=AG; Enzymes=EZY; Additives=ADD; B12 (µg)=B12
Use=USE; Serving=SG; Degree of evidence(Australian Institute of Sport)=DE; Certification=CT; Frequency=FC; Source of information=SI
✓ Complies with what is established in the legislation.

PR	CH	KCAL	PROT	AA	BCAA	HC	SUG	FAT	NA
Whey isolate protein vanilla flavor	Named sport	366	87	Alanine 4,41<br>Arginine 1,72<br>Aspartic acid 9,19<br>Cysteine 2.01<br>Aspartic acid 15,43<br>Glycine 1,31<br>Histidine 1,31<br>Isoleucine 6<br>Leucine 8.62<br>Lysine 8,23<br>Methionine 1,84<br>Phenylalanine 2,46<br>Proline 5,18<br>Serine 4,13<br>Threonine 6.01<br>Tryptophan 1,35<br>Tyrosine 2.29<br>Valine 5.51	20,13	2,6	1,3 g	0,9	200

*Does not comply with the Regulation.

Screening 1					Screening 2		Screening 3				
R1	R2	R3	R4	R5	AP	LA	OR	AG	EZY	ADD	B12
✓	✓	"Essential for recovery and muscle mass gain" *	No claims	No claims	27,19	No claims	No claims	Milk, soy	No claims	Sucralose<br>Salt<br>Fenugreek<br>Vitamin B6<br>Vitamin B2<br>Vitamin B1	No claims

SG	DE	CT	FC	SI
31,25	A	No	No claims	Label

Abbreviations: Product(100gr)=PR; Comercial House=CH; Kilocalories=Kcal; Protein(g)=PROT; Aminogram(g)=AA; BCAA(g)=BCAA; Carbohydrates(g)=HC; Of which sugars(g)=SUG; Fats(g)=FAT; Sodium(mg)=NA

Screening 1: Regulation 1169/2011=R1; Royal Decree 1487/2009=R2; Regulation 432/2012=R3; World Anti-Doping Code=R5

Screening 2: Amount of protein recommended dose=AP; Limiting amino acid=LA

Screening 3: Others manufacture's intake recommendation=OR; Allergens=AG; Enzymes=EZY; Additives=ADD; B12 (µg)=B12

Use=USE; Serving=SG; Degree of evidence(Australian Institute of Sport)=DE; Certification=CT; Frequency=FC; Source of information=SI

✓ Complies with what is established in the legislation.

PR	CH	KCAL	PROT	AA	BCAA	HC	SUG	FAT	NA
Whey protein concentrate and isolate	The power of nutrition	380	73	Alanine 3,508<br>Arginine 2,013<br>Aspartic acid 8,044<br>Cysteine 1,850<br>Glutamic acid 1,2813<br>Glycine 1,254<br>Histidine 1,457<br>Isoleucine 4,017<br>Leucine 8,574<br>Lysine 7,236<br>Methionine 1,655<br>Phenylalanine 2,579<br>Proline 3,596<br>Serine 3,253<br>Threonine 3,911<br>Tyrosine 2,437<br>Valine 3,735<br>Tryptophan 1,36	16,326	7,5	5,5 g	6,1	720

SG	DE	CT	FC	SI
30 g	A	No	No claims	Label

Screening 1					Screening 2		Screening 3				
R1	R2	R3	R4	R5	AP	LA	OR	AG	EZY	ADD	B12
No claims	No claims	No claims	No claims	No claims	21,9	No claims	No claims	Milk, soy	Amylase<br>Protease<br>Lactase<br>Lipase<br>Cellulase	Sunflower lecithin<br>Soy lecithin<br>Acacia gum and xanthan<br>Aroma<br>Silica anti-caking substance<br>Calcium phosphate anti-caking substance<br>Sodium chloride<br>Sucralose<br>Steviol glycosides<br>Wheat fiber<br>Colostrum	No claims

Abbreviations: Product(100gr)=PR; Comercial House=CH; Kilocalories=Kcal; Protein(g)=PROT; Aminogram(g)=AA; BCAA(g)=BCAA; Carbohydrates(g)=HC; Of which sugars(g)=SUG; Fats(g)=FAT; Sodium(mg)=NA
Screening 1: Regulation 1169/2011=R1; Royal Decree 1487/2009=R2; Regulation 432/2012=R3; World Anti-Doping Code=R5
Screening 2: Amount of protein recommended dose=AP; Limiting amino acid=LA
Screening 3: Others manufacture's intake recommendation=OR; Allergens=AG; Enzymes=EZY; Additives=ADD; B12 (µg)=B12
Use=USE; Serving=SG; Degree of evidence(Australian Institute of Sport)=DE; Certification=CT; Frequency=FC; Source of information=SI
✓ Complies with what is established in the legislation.

PR	CH	KCAL	PROT	AA	BCAA	HC	SUG	FAT	NA
Whey protein isolate, hydrolysate and concentrate	Blue lab	370	70	Alanine 1,2<br>Arginine 0.6<br>Aspartic acid 2,6<br>Cysteine 0,3<br>Glutamic acid 4<br>Glycine 0.5<br>Histidine 0,4<br>Isoleucine 1,4<br>Leucine 2.4<br>Lysine 2,1<br>Methionine 0,4<br>Phenylalanine 0.7<br>Proline 1,4<br>Serine 1,2<br>Threonine 1,6<br>Tryptophan 0.4<br>Tyrosine 0.7<br>Valine 1,3	5,13	6,1	4,6 g	5,5	560

SG	DE	CT	FC	SI
34 g	A	No	No claims	Label

Screening 1					Screening 2		Screening 3				
R1	R2	R3	R4	R5	AP	LA	OR	AG	EZY	ADD	B12
✓	✓	No claims	No claims	No claims	23,8	No claims	No claims	Milk, soy and it is possible that contains sesame, egg, tree nuts, peanuts, fish/crustaceans/shellfish oils and wheat products	No Claims	Cocoa powder<br>Inulin<br>Aroma<br>Sodium chloride<br>Guar and xanthan gum<br>Sucralose<br>Tolerase (lactase at a stable pH)	8,29

Abbreviations: Product(100gr)=PR; Comercial House=CH; Kilocalories=Kcal; Protein(g)=PROT; Aminogram(g)=AA; BCAA(g)=BCAA; Carbohydrates(g)=HC; Of which sugars(g)=SUG; Fats(g)=FAT; Sodium(mg)=NA

Screening 1: Regulation 1169/2011=R1; Royal Decree 1487/2009=R2; Regulation 432/2012=R3; World Anti-Doping Code=R5

Screening 2: Amount of protein recommended dose=AP; Limiting amino acid=LA

Screening 3: Others manufacture's intake recommendation=OR; Allergens=AG; Enzymes=EZY; Additives=ADD; B12 (µg)=B12

Use=USE; Serving=SG; Degree of evidence(Australian Institute of Sport)=DE; Certification=CT; Frequency=FC; Source of information=SI

✓ Complies with what is established in the legislation.

PR	CH	KCAL	PROT	AA	BCAA	HC	SUG	FAT	NA
Whey protein concentrate and isolate, strawberry and cherry flavored egg protein	M double you	354	15	Isoleucine 5,6<br>Leucine 9.9<br>Valine 6<br>Phenylalanine 3,8<br>Lysine 8,7<br>Methionine 2,5<br>Threonine 5.7<br>Tryptophan 1,4<br>Arginine 3<br>Histidine 2,1<br>Alanine 4,3<br>Aspartic acid 9,2<br>Cysteine 1,6<br>Glutamic acid 18,5<br>Proline 7,1<br>Serine 5.1<br>Tyrosine 3.7<br>Glycine 1,8	21,5	36	3,4 g	0,4	92

SG	DE	CT	FC	SI
50 g	A	No	No claims	Label

Screening 1					Screening 2		Screening 3				
R1	R2	R3	R4	R5	AP	LA	OR	AG	EZY	ADD	B12
No claims	No claims	No claims	No claims	No claims	7,5	No claims	No claims	Milk, soy	Hydrolase Fructose	Maltodextrin<br>Corn starch<br>Soy lecithin<br>Aromas (strawberry and cherry)<br>Calcium caseinate<br>Inulin<br>Anhydrous citric acid<br>Red dye<br>Sodium chloride<br>Xanthan gum<br>Sucralose<br>L-leucine<br>L-isoleucine<br>L-valine	No Claims

Abbreviations: Product(100gr)=PR; Comercial House=CH; Kilocalories=Kcal; Protein(g)=PROT; Aminogram(g)=AA; BCAA(g)=BCAA; Carbohydrates(g)=HC; Of which sugars(g)=SUG; Fats(g)=FAT; Sodium(mg)=NA
Screening 1: Regulation 1169/2011=R1; Royal Decree 1487/2009=R2; Regulation 432/2012=R3; World Anti-Doping Code=R5
Screening 2: Amount of protein recommended dose=AP; Limiting amino acid=LA
Screening 3: Others manufacture's intake recommendation=OR; Allergens=AG; Enzymes=EZY; Additives=ADD; B12 (μg)=B12
Use=USE; Serving=SG; Degree of evidence(Australian Institute of Sport)=DE; Certification=CT; Frequency=FC; Source of information=SI
✓Complies with what is established in the legislation.

PR	CH	KCAL	PROT	AA	BCAA	HC	SUG	FAT	NA
Hydrolyzed whey protein	Nutrend	382	76,8	Valine 4,397<br>Tyrosine 2,332<br>Tryptophan 1,078<br>Threonine 5,567<br>Serine 4,082<br>Proline 4,355<br>Phenylalanine 2,472<br>Methionine 1,393<br>Lysine 6,904<br>Leucine 8,081<br>Isoleucine 4,355<br>Histidine 1,393<br>Glycine 1,425<br>Glutamic acid 13,15<br>Cysteine 1,842<br>Aspartic acid 8,214<br>Arginine 2,066<br>Alanine 3,683	16,833	9,63 g		5,9	No claims

*Does not comply with the Regulation.

SG	DE	CT	FC	SI
30 g	A	No		Label

Screening 1					Screening 2		Screening 3				
R1	R2	R3	R4	R5	AP	LA	OR	AG	EZY	ADD	B12
✓	✓	"Recover after training" *	No claims	No claims	23,04	No claims	No claims	Lactose, soy	Digestive enzyme blend	Hydrogenated calcium phosphate<br>Microcrystalline cellulose<br>Silicon dioxide<br>Magnesium stearate<br>Hydroxylmethyl-proplpilcellulose<br>Talc	No claims

Abbreviations: Product(100gr)=PR; Comercial House=CH; Kilocalories=Kcal; Protein(g)=PROT; Aminogram(g)=AA; BCAA(g)=BCAA; Carbohydrates(g)=HC; Of which sugars(g)=SUG; Fats(g)=FAT; Sodium(mg)=NA
Screening 1: Regulation 1169/2011=R1; Royal Decree 1487/2009=R2; Regulation 432/2012=R3; World Anti-Doping Code=R5
Screening 2: Amount of protein recommended dose=AP; Limiting amino acid=LA
Screening 3: Others manufacture's intake recommendation=OR; Allergens=AG; Enzymes=EZY; Additives=ADD; B12 (μg)=B12
Use=USE; Serving=SG; Degree of evidence(Australian Institute of Sport)=DE; Certification=CT; Frequency=FC; Source of information=SI
✓ Complies with what is established in the legislation.

PR	CH	KCAL	PROT	AA	BCAA	HC	SUG	FAT	NA
Hydrolyzed protein isolate and vanilla flavored whey protein isolate	Dymatize Iso 100	366	82	No claims	No claims	7,6	1,6 g	0,4	340

SG	DE	CT	FC	SI
30g	A	No		Label

Screening 1					Screening 2		Screening 3				
R1	R2	R3	R4	R5	AP	LA	OR	AG	EZY	ADD	B12
✓	✓	No claims	No claims	No claims	24,6	No claims	No claims	Milk, soy	No claims	Aroma<br>Lecithins<br>Salt<br>Sucralose<br>Steviol glycosidesl	No claims

Abbreviations: Product(100gr)=PR; Comercial House=CH; Kilocalories=Kcal; Protein(g)=PROT; Aminogram(g)=AA; BCAA(g)=BCAA; Carbohydrates(g)=HC; Of which sugars(g)=SUG; Fats(g)=FAT; Sodium(mg)=NA

Screening 1: Regulation 1169/2011=R1; Royal Decree 1487/2009=R2; Regulation 432/2012=R3; World Anti-Doping Code=R5

Screening 2: Amount of protein recommended dose=AP; Limiting amino acid=LA

Screening 3: Others manufacture's intake recommendation=OR; Allergens=AG; Enzymes=EZY; Additives=ADD; B12 (μg)=B12

Use=USE; Serving=SG; Degree of evidence(Australian Institute of Sport)=DE; Certification=CT; Frequency=FC; Source of information=SI

✓ Complies with what is established in the legislation.

PR	CH	KCAL	PROT	AA	BCAA	HC	SUG	FAT	NA
Protein whey isolate flavor cookies and creams	7nutrition	363	85	Glutamic acid 14,692<br>Lysine 9,692<br>Leucine 9,097<br>Aspartic acid 8,842<br>Threonine 5,356<br>Isoleucine 5,185<br>Valine 4,676<br>Proline 4,591<br>Alanine 4,336<br>Serine 3,656<br>Phenylalanine 2,55<br>Tyrosine 2,466<br>Cysteine 2,126<br>Arginine 1,955<br>Methionine 1,871<br>Histidine 1,445<br>Glycine 1,445<br>Tryptophan 1,021	18,058	2,5	2,2 g	1,4	120

SG	DE	CT	FC	SI
30 g	A	No	No claims	Label

Screening 1					Screening 2		Screening 3				
R1	R2	R3	R4	R5	AP	LA	OR	AG	EZY	ADD	B12
✓	✓	No claims	No claims	No claims	25,5	No claims	No claims	Milk, soy, nuts	No claims	Cocoa<br>Aromas<br>Gum arabic<br>Malic acid<br>Sucralose	No claims

Abbreviations: Product(100gr)=PR; Comercial House=CH; Kilocalories=Kcal; Protein(g)=PROT; Aminogram(g)=AA; BCAA(g)=BCAA; Carbohydrates(g)=HC; Of which sugars(g)=SUG; Fats(g)=FAT; Sodium(mg)=NA

Screening 1: Regulation 1169/2011=R1; Royal Decree 1487/2009=R2; Regulation 432/2012=R3; World Anti-Doping Code=R5

Screening 2: Amount of protein recommended dose=AP; Limiting amino acid=LA

Screening 3: Others manufacture's intake recommendation=OR; Allergens=AG; Enzymes=EZY; Additives=ADD; B12 (µg)=B12

Use=USE; Serving=SG; Degree of evidence(Australian Institute of Sport)=DE; Certification=CT; Frequency=FC; Source of information=SI

✓ Complies with what is established in the legislation.

PR	CH	KCAL	PROT	AA	BCAA	HC	SUG	FAT	NA
Gold Whey chocolate and hazelnut flavor (hydrolyzed collagen and whey protein)	FullGas	379	74	Aspartic acid 11<br>Glutamic acid 18,1<br>Alanine 5<br>Arginine 2,1<br>Cysteine 2,2<br>Phenylalanine 3<br>Glycine 1,4<br>Histidine 1,7<br>Isoleucine 6,4<br>Leucine 10.6<br>Lysine 9,6<br>Methionine 2,2<br>Proline 5.5<br>Serine 4.6<br>Tyrosine 2.6<br>Threonine 6.7<br>Tryptophan 1,4<br>Valine 5,9<br>Hydroxyvaline 0,39<br>Hydroxyproline 4,21	22,9	6,3	2,9 g	6	316

Combined whey protein with other products.

SG	DE	CT	FC	SI
40 g	A	No	No claims	Web commercial house

Screening 1					Screening 2		Screening 3				
R1	R2	R3	R4	R5	AP	LA	OR	AG	EZY	ADD	B12
No claims	✓	No claims	No claims	No claims	29,6	No claims	No claims	Dairy, soy	No claims	Degreased cocoa powder<br>Aroma<br>Sucralose<br>Soy lecithin<br>Guar and xanthan gum	No claims

Abbreviations: Product(100gr)=PR; Comercial House=CH; Kilocalories=Kcal; Protein(g)=PROT; Aminogram(g)=AA; BCAA(g)=BCAA; Carbohydrates(g)=HC; Of which sugars(g)=SUG; Fats(g)=FAT; Sodium(mg)=NA
Screening 1: Regulation 1169/2011=R1; Royal Decree 1487/2009=R2; Regulation 432/2012=R3; World Anti-Doping Code=R5
Screening 2: Amount of protein recommended dose=AP; Limiting amino acid=LA
Screening 3: Others manufacture's intake recommendation=OR; Allergens=AG; Enzymes=EZY; Additives=ADD; B12 (µg)=B12
Use=USE; Serving=SG; Degree of evidence(Australian Institute of Sport)=DE; Certification=CT; Frequency=FC; Source of information=SI
✓ Complies with what is established in the legislation.

PR	CH	KCAL	PROT	AA	BCAA	HC	SUG	FAT	NA
Gold Whey double chocolate flavor (hydrolyzed collagen and whey protein)	FullGas	381	75	Aspartic acid 8,68<br>Glutamic acid 14,75<br>Alanine 6.69<br>Arginine 4,64<br>Cysteine 1,2<br>Phenylalanine 2,83<br>Glycine 10,73<br>Histidine 1,27<br>Isoleucine 4,29<br>Leucine 7.36<br>Lysine 7.03<br>Methionine 1,5<br>Proline 9,13<br>Serine 4,25<br>Tyrosine 1.83<br>Threonine 4,75<br>Tryptophan 1,14<br>Valine 4,51<br>Hydroxylysine 0,48<br>Hydroxyproline 5,18	16,16	5,3	3,4 g	6	324

Combined whey protein with other products.

SG	DE	CT	FC	SI
40 g	A	No	No claims	Web commercial house

Screening 1					Screening 2		Screening 3				
R1	R2	R3	R4	R5	AP	LA	OR	AG	EZY	ADD	B12
No claims	No claims	✓	No claims	No claims	30	No claims	No claims	Dairy, soy	No claims	Degreased cocoa powder<br>Aroma<br>Sucralose<br>Soy lecithin<br>Guar and xanthan gum	No claims

Abbreviations: Product(100gr)=PR; Comercial House=CH; Kilocalories=Kcal; Protein(g)=PROT; Aminogram(g)=AA; BCAA(g)=BCAA; Carbohydrates(g)=HC; Of which sugars(g)=SUG; Fats(g)=FAT; Sodium(mg)=NA
Screening 1: Regulation 1169/2011=R1; Royal Decree 1487/2009=R2; Regulation 432/2012=R3; World Anti-Doping Code=R5
Screening 2: Amount of protein recommended dose=AP; Limiting amino acid=LA
Screening 3: Others manufacture's intake recommendation=OR; Allergens=AG; Enzymes=EZY; Additives=ADD; B12 (µg)=B12
Use=USE; Serving=SG; Degree of evidence(Australian Institute of Sport)=DE; Certification=CT; Frequency=FC; Source of information=SI
✓ Complies with what is established in the legislation.

PR	CH	KCAL	PROT	AA	BCAA	HC	SUG	FAT	NA
Protein and colostrum flavor yogurt and lemon (milk protein isolate and collagen hydrolysate)	FullGas	368	85	Aspartic acid 9,16<br>Glutamic acid 15,29<br>Alanine 6,85<br>Arginine 4,27<br>Cysteine 1,53<br>Phenylalanine 2,85<br>Glycine 9,77<br>Histidine 1,29<br>Isoleucine 4.84<br>Leucine 8,11<br>Lysine 7,84<br>Methionine 1,84<br>Proline 9,36<br>Serine 4.42<br>Tyrosine 2.05<br>Threonine 5,36<br>Tryptophan 1,14<br>Valine 4.78<br>Hydroxylysine 0.43<br>Hydroxyproline 4.64	17,73	3,4	0,9 g	1,3	440

Combined whey protein with other products.

*Does not comply with the Regulation.

SG	DE	CT	FC	SI
40 g	A	No	No claims	Web commercial house

Screening 1					Screening 2		Screening 3				
R1	R2	R3	R4	R5	AP	LA	OR	AG	EZY	ADD	B12
No clais	No claims	✓	No claims	Contains colostrum *	35	No claims	No claims	Dairy, soy	Complex enzymatic	Soy lecithin<br>Aromas<br>Guar gum<br>Xanthan gum<br>Sucralose	No claims

Abbreviations: Product(100gr)=PR; Comercial House=CH; Kilocalories=Kcal; Protein(g)=PROT; Aminogram(g)=AA; BCAA(g)=BCAA; Carbohydrates(g)=HC; Of which sugars(g)=SUG; Fats(g)=FAT; Sodium(mg)=NA
Screening 1: Regulation 1169/2011=R1; Royal Decree 1487/2009=R2; Regulation 432/2012=R3; World Anti-Doping Code=R5
Screening 2: Amount of protein recommended dose=AP; Limiting amino acid=LA
Screening 3: Others manufacture's intake recommendation=OR; Allergens=AG; Enzymes=EZY; Additives=ADD; B12 (µg)=B12
Use=USE; Serving=SG; Degree of evidence(Australian Institute of Sport)=DE; Certification=CT; Frequency=FC; Source of information=SI
✓ Complies with what is established in the legislation.

CASEIN

PR	CH	KCAL	PROT	AA	BCAA	HC	SUG	FAT	NA
Chocolate flavored casein	My protein	356	76	No claims	No claims	4	4 g	1,5	100

SG	DE	CT	FC	SI
30 g	A	No	No claims	Web commercial house

Screening 1					Screening 2		Screening 3				
R1	R2	R3	R4	R5	AP	LA	OR	AG	EZY	ADD	B12
✓	✓	"Growth and maintenance of muscle mass" ✓	✓	No claims	22,8	No claims	30 minutes before sleeping	No claims	No claims	Cocoa powder reduced in fat<br>Flavoring<br>Sucralose<br>Soy lecithin	No claims

Abbreviations: Product(100gr)=PR; Comercial House=CH; Kilocalories=Kcal; Protein(g)=PROT; Aminogram(g)=AA; BCAA(g)=BCAA; Carbohydrates(g)=HC; Of which sugars(g)=SUG; Fats(g)=FAT; Sodium(mg)=NA
Screening 1: Regulation 1169/2011=R1; Royal Decree 1487/2009=R2; Regulation 432/2012=R3; World Anti-Doping Code=R5
Screening 2: Amount of protein recommended dose=AP; Limiting amino acid=LA
Screening 3: Others manufacture's intake recommendation=OR; Allergens=AG; Enzymes=EZY; Additives=ADD; B12 (μg)=B12
Use=USE; Serving=SG; Degree of evidence(Australian Institute of Sport)=DE; Certification=CT; Frequency=FC; Source of information=SI
✓ Complies with what is established in the legislation.

PR	CH	KCAL	PROT	AA	BCAA	HC	SUG	FAT	NA
Milk protein isolate double chocolate flavor (iso micellar casein)	FullGas	345	73	Aspartic acid 7,1<br>Glutamic acid 20,74<br>Alanine 3,01<br>Arginine 3,04<br>Cysteine 0.57<br>Phenylalanine 4,6<br>Glycine 1,75<br>Histidine 2,73<br>Isoleucine 4.95<br>Leucine 9.27<br>Lysine 7,94<br>Methionine 2,47<br>Proline 10.55<br>Serine 5.03<br>Tyrosine 4.42<br>Threonine 4.02<br>Tryptophan 1.06<br>Valine 6.07	20,29	4	1,7 g	3,1	140

SG	DE	CT	FC	SI
40 g	A	No	No claims	Web commercial house

Screening 1					Screening 2		Screening 3				
R1	R2	R3	R4	R5	AP	LA	OR	AG	EZY	ADD	B12
✓	✓	No claims	No claims	No claims	29,2	No claims	On weight loss diets or before bed	Milk, soy	No claims	Degreased cocoa powder<br>Aromas<br>Sucralose	No claims

Abbreviations: Product(100gr)=PR; Comercial House=CH; Kilocalories=Kcal; Protein(g)=PROT; Aminogram(g)=AA; BCAA(g)=BCAA; Carbohydrates(g)=HC; Of which sugars(g)=SUG; Fats(g)=FAT; Sodium(mg)=NA
Screening 1: Regulation 1169/2011=R1; Royal Decree 1487/2009=R2; Regulation 432/2012=R3; World Anti-Doping Code=R5
Screening 2: Amount of protein recommended dose=AP; Limiting amino acid=LA
Screening 3: Others manufacture's intake recommendation=OR; Allergens=AG; Enzymes=EZY; Additives=ADD; B12 (µg)=B12
Use=USE; Serving=SG; Degree of evidence(Australian Institute of Sport)=DE; Certification=CT; Frequency=FC; Source of information=SI
✓ Complies with what is established in the legislation.

PR	CH	KCAL	PROT	AA	BCAA	HC	SUG	FAT	NA
Milk protein isolate double chocolate flavor (iso micellar casein) strawberry flavor	Nutrend	351	76,9	Valine 3,854<br>Tyrosine 2,698<br>Tryptophan 1,698<br>Threonine 3,931<br>Serine 3,468<br>Proline 4,008<br>Phenylalanine 2,852<br>Methionine 1,696<br>Lysine 6,904<br>Leucine 9,326<br>Isoleucine 4,239<br>Histidine 1,541<br>Glycine 1,388<br>Glutamic acid and glutamine 12,795<br>Cysteine 2,081<br>Aspartic acid 8,401<br>Arginine 2,081<br>Alanine 3,7	17,419	6,2	4 g	1,7	80

SG	DE	CT	FC	SI
30g	A	No	No claims	Label

Screening 1					Screening 2		Screening 3				
R1	R2	R3	R4	R5	AP	LA	OR	AG	EZY	ADD	B12
✓	✓	No claims	No claims	No claims	23,07		Slimming diets	Milk	No claims	Aromas<br>Wheat fibers<br>Acacia gum and xanthan<br>Soy lecithin<br>Beet red concentrate<br>Silicon dioxide<br>Steviol sucralose and glycosides	No claims

Abbreviations: Product(100gr)=PR; Comercial House=CH; Kilocalories=Kcal; Protein(g)=PROT; Aminogram(g)=AA; BCAA(g)=BCAA; Carbohydrates(g)=HC; Of which sugars(g)=SUG; Fats(g)=FAT; Sodium(mg)=NA
Screening 1: Regulation 1169/2011=R1; Royal Decree 1487/2009=R2; Regulation 432/2012=R3; World Anti-Doping Code=R5
Screening 2: Amount of protein recommended dose=AP; Limiting amino acid=LA
Screening 3: Others manufacture's intake recommendation=OR; Allergens=AG; Enzymes=EZY; Additives=ADD; B12 (μg)=B12
Use=USE; Serving=SG; Degree of evidence(Australian Institute of Sport)=DE; Certification=CT; Frequency=FC; Source of information=SI
✓ Complies with what is established in the legislation.

PR	CH	KCAL	PROT	AA	BCAA	HC	SUG	FAT	NA
Micellar casein, concentrated and hydrolyzed vanilla flavored protein	7nutrition	368	72	Glutamic acid 14,674<br>Proline 6,852<br>Leucine 6,748<br>Lysine 5,776<br>Aspartic acid 5,206<br>Valine 4,438<br>Serine 3,754<br>Isoleucine 3,629<br>Tyrosine 3,456<br>Phenylalanine 3,271<br>Threonine 3,201<br>Alanine 2,337<br>Arginine 2269<br>Methionine 1,9<br>Histidine 1,855<br>Glycine 1,229<br>Cysteine 0.523<br>Tryptophan 0.882	17,815	13,8	7.2 g sugar 4.6 g dextrose	2,8	40

SG	DE	CT	FC	SI
30 g	A	No	1-2 times a day	Label

Screening 1					Screening 2		Screening 3				
R1	R2	R3	R4	R5	AP	LA	OR	AG	EZY	ADD	B12
✓	✓	No claims	No claims	No claims	21,6		No claims	Milk, soy, may contain traces of nuts and eggs	No claims	Dextrose<br>E414<br>Soy lecithin<br>Aromas<br>Sucralose	No claims

Abbreviations: Product(100gr)=PR; Comercial House=CH; Kilocalories=Kcal; Protein(g)=PROT; Aminogram(g)=AA; BCAA(g)=BCAA; Carbohydrates(g)=HC; Of which sugars(g)=SUG; Fats(g)=FAT; Sodium(mg)=NA
Screening 1: Regulation 1169/2011=R1; Royal Decree 1487/2009=R2; Regulation 432/2012=R3; World Anti-Doping Code=R5
Screening 2: Amount of protein recommended dose=AP; Limiting amino acid=LA
Screening 3: Others manufacture's intake recommendation=OR; Allergens=AG; Enzymes=EZY; Additives=ADD; B12 (µg)=B12
Use=USE; Serving=SG; Degree of evidence(Australian Institute of Sport)=DE; Certification=CT; Frequency=FC; Source of information=SI
✓ Complies with what is established in the legislation.

PR	CH	KCAL	PROT	AA	BCAA	HC	SUG	FAT	NA
Micellar casein chocolate flavor	Real pharm	346,8	76	Leucine 10.6<br>Isoleucine 6,4<br>Valine 5.9<br>Total BCAA 22.9<br>Aspartic acid 11<br>Glutamic acid 18,1<br>Serine 4.6<br>Glycine 1,4<br>Histidine 1,7<br>Arginine 2,1<br>Threonine 6.7<br>Alanine 5<br>Proline 5.5<br>Tyrosine 2.6<br>Methionine 2,2<br>Cysteine 2,2<br>Phenylalanine 3<br>Lysine 9,6<br>Tryptophan 1,4	22,9	6	5 g	2	200

SG	DE	CT	FC	SI
30,4 g	A	No	No claims	Label

Screening 1					Screening 2		Screening 3				
R1	R2	R3	R4	R5	AP	LA	OR	AG	EZY	ADD	B12
✓	✓	No claims	No claims	No claims	23,104	No claims	No claims	Milk	No claims	Cocoa<br>Aroma<br>Citric acid<br>(strawberry flavor)<br>Carboxymethylcellulose<br>Sucralose<br>Dyes	No claims

Abbreviations: Product(100gr)=PR; Comercial House=CH; Kilocalories=Kcal; Protein(g)=PROT; Aminogram(g)=AA; BCAA(g)=BCAA; Carbohydrates(g)=HC; Of which sugars(g)=SUG; Fats(g)=FAT; Sodium(mg)=NA
Screening 1: Regulation 1169/2011=R1; Royal Decree 1487/2009=R2; Regulation 432/2012=R3; World Anti-Doping Code=R5
Screening 2: Amount of protein recommended dose=AP; Limiting amino acid=LA
Screening 3: Others manufacture's intake recommendation=OR; Allergens=AG; Enzymes=EZY; Additives=ADD; B12 (µg)=B12
Use=USE; Serving=SG; Degree of evidence(Australian Institute of Sport)=DE; Certification=CT; Frequency=FC; Source of information=SI
✓ Complies with what is established in the legislation.

EGG ALBUMIN

PR	CH	KCAL	PROT	AA	BCAA	HC	SUG	FAT	NA
Hightly concentrate protein	Prozis	99,5	24	Alanine 1,52<br>Arginine 1,31<br>Aspartic acid 2,67<br>Cystine 0,65<br>Glutamic acid 3,28<br>Glycine 0,90<br>Histidine 0,15<br>Isoleucine 1,09<br>Leucine 1,95<br>Lysine 1,43<br>Methionine 0,91<br>Phenylalanine 1,30<br>Proline 1,22<br>Serine 1,72<br>Threonine 1,10<br>Tryptophan 0,40<br>Tyrosine 0,55<br>Valine 1,4	4,44	0,9	0	0	280

SG	DE	CT	FC	SI
30 g	A	No claims	Take a maximum of 4 doses a day	Web commercial house

Screening 1					Screening 2		Screening 3				
R1	R2	R3	R4	R5	AP	LA	OR	AG	EZY	ADD	B12
✓	✓	No claims	No claims	No claims	7,2	No claims	No claims	Eggs, it is possible that contain traces of milk, soy and gluten	No claims	No claims	No claims

Abbreviations: Product(100gr)=PR; Comercial House=CH; Kilocalories=Kcal; Protein(g)=PROT; Aminogram(g)=AA; BCAA(g)=BCAA; Carbohydrates(g)=HC; Of which sugars(g)=SUG; Fats(g)=FAT; Sodium(mg)=NA

Screening 1: Regulation 1169/2011=R1; Royal Decree 1487/2009=R2; Regulation 432/2012=R3; World Anti-Doping Code=R5

Screening 2: Amount of protein recommended dose=AP; Limiting amino acid=LA

Screening 3: Others manufacture's intake recommendation=OR; Allergens=AG; Enzymes=EZY; Additives=ADD; B12 (µg)=B12

Use=USE; Serving=SG; Degree of evidence(Australian Institute of Sport)=DE; Certification=CT; Frequency=FC; Source of information=SI

✓ Complies with what is established in the legislation.

PR	CH	KCAL	PROT	AA	BCAA	HC	SUG	FAT	NA
Egg white powder	Myprotein	336	78		No claims	5,3	0	0,3	7500

Screening 1					Screening 2		Screening 3				
R1	R2	R3	R4	R5	AP	LA	OR	AG	EZY	ADD	B12
✓	✓	"Proteins contribute to the increase and maintenance of muscle mass"; "Creatine has been shown to improve physical performance in short-burst, high-intensity exercise. Beneficial effects are obtained with a daily intake of 3 g of creatine" ✓	No claims	No claims	23,4	No claims	Take 30 minutes before or after training and preferably take for breakfast	Eggs	No claims	No claims	No claims

SG	DE	CT	FC	SI
30 g	A			Web commercial house

Abbreviations: Product(100gr)=PR; Comercial House=CH; Kilocalories=Kcal; Protein(g)=PROT; Aminogram(g)=AA; BCAA(g)=BCAA; Carbohydrates(g)=HC; Of which sugars(g)=SUG; Fats(g)=FAT; Sodium(mg)=NA
Screening 1: Regulation 1169/2011=R1; Royal Decree 1487/2009=R2; Regulation 432/2012=R3; World Anti-Doping Code=R5
Screening 2: Amount of protein recommended dose=AP; Limiting amino acid=LA
Screening 3: Others manufacture's intake recommendation=OR; Allergens=AG; Enzymes=EZY; Additives=ADD; B12 (μg)=B12
Use=USE; Serving=SG; Degree of evidence(Australian Institute of Sport)=DE; Certification=CT; Frequency=FC; Source of information=SI
✓ Complies with what is established in the legislation.

PR	CH	KCAL	PROT	AA	BCAA	HC	SUG	FAT	NA
Egg white powder	The protein Works	336	78,2	No claims	No claims	5,3	0	0,3	7500

Screening 1					Screening 2		Screening 3				
R1	R2	R3	R4	R5	AP	LA	OR	AG	EZY	ADD	B12
✓	✓	No claims	No claims	No claims	23,46	No claims	No claims	Eggs	No claims	No claims	No claims

SG	DE	CT	FC	SI
30 g	A	No claims	2-3 times a day	Web commercial house

Abbreviations: Product(100gr)=PR; Comercial House=CH; Kilocalories=Kcal; Protein(g)=PROT; Aminogram(g)=AA; BCAA(g)=BCAA; Carbohydrates(g)=HC; Of which sugars(g)=SUG; Fats(g)=FAT; Sodium(mg)=NA
Screening 1: Regulation 1169/2011=R1; Royal Decree 1487/2009=R2; Regulation 432/2012=R3; World Anti-Doping Code=R5
Screening 2: Amount of protein recommended dose=AP; Limiting amino acid=LA
Screening 3: Others manufacture's intake recommendation=OR; Allergens=AG; Enzymes=EZY; Additives=ADD; B12 (µg)=B12
Use=USE; Serving=SG; Degree of evidence(Australian Institute of Sport)=DE; Certification=CT; Frequency=FC; Source of information=SI
✓ Complies with what is established in the legislation.

SOY

PR	CH	KCAL	PROT	AA	BCAA	HC	SUG	FAT	NA
Instant cocoa flavor protein (soy protein)	Santiveri	400	90	Arginine 6.85 Histidine 2.44 Isoleucine 4.84 Leucine 7.93 Methionine 1.38 Phenylalanine 5.29 Threonine 3.81 Tryptophan 1.08 Valine 4.9 Serine 5.04 Glutamic acid 16,38 Alanine 4.14 Glycine 3.78 Proline 4.77 Aspartic acid 9,9 Tyrosine 3.5 Cystine 1.08 Lysine 5,71	17,7	2,3	0,5 g	3,5	1160

SG	DE	CT	FC	SI
30 g	A	No	No claims	Label

Screening 1					Screening 2		Screening 3				
R1	R2	R3	R4	R5	AP	LA	OR	AG	EZY	ADD	B12
✓	✓	"Contributes to the increase and conservation of muscle mass" ✓	✓	No claims	27	No claims	During intense sports practice or after competiti on	Soy and sulphites	No claims	Cyclamate Saccharin Aroma Sulfites (naturally present in soy protein isolate) Vitamins: C, niacin, pantothenic acid, riboflavin, thiamine, B6, A, folic acid, biotin, K, D and B12	5

Abbreviations: Product(100gr)=PR; Comercial House=CH; Kilocalories=Kcal; Protein(g)=PROT; Aminogram(g)=AA; BCAA(g)=BCAA; Carbohydrates(g)=HC; Of which sugars(g)=SUG; Fats(g)=FAT; Sodium(mg)=NA
Screening 1: Regulation 1169/2011=R1; Royal Decree 1487/2009=R2; Regulation 432/2012=R3; World Anti-Doping Code=R5
Screening 2: Amount of protein recommended dose=AP; Limiting amino acid=LA
Screening 3: Others manufacture's intake recommendation=OR; Allergens=AG; Enzymes=EZY; Additives=ADD; B12 (μg)=B12
Use=USE; Serving=SG; Degree of evidence(Australian Institute of Sport)=DE; Certification=CT; Frequency=FC; Source of information=SI
✓ Complies with what is established in the legislation.

OTHER PLANT PROTEINS

PR	CH	KCAL	PROT	AA	BCAA	HC	SUG	FAT	NA
Vegan protein (pea and rice isolate)	Life pro	436,24	76,91	L-Leucine 7,53<br>L-Isoleucine 3,94<br>L-Valine 4,42<br>L-Aspartic Acid 9,79<br>L-glutamic acid 16,71<br>L-Serine 4,63<br>L-Glycine 3,47<br>L-histidine 2,15<br>L-Arginine 7,52<br>L-Threonine 3,24<br>L-Alanine 3,61<br>L-Proline 3,54<br>L-Tyrosine 3,52<br>L-Methionine 1,26<br>L-Cysteine 1<br>L-Phenylalanine 4,78<br>L-Lysine 3,75<br>L-Tryptophan 0.77	18,14	12,49	0,15 g	7,18	248

*Does not comply with the Regulation.

SG	DE	CT	FC	SI
30 g	A	No	1-2 times a day	Web commercial house

Screening 1					Screening 2		Screening 3				
R1	R2	R3	R4	R5	AP	LA	OR	AG	EZY	ADD	B12
✓	✓	"Accelerates the recovery process and prevents muscle catabolism" *	No claims	No claims	21,073	No claims	Before or after training due to its rapid absorption	May contain milk, soy, egg, and gluten	No claims	Sucralose, aromas	No claims

Abbreviations: Product(100gr)=PR; Comercial House=CH; Kilocalories=Kcal; Protein(g)=PROT; Aminogram(g)=AA; BCAA(g)=BCAA; Carbohydrates(g)=HC; Of which sugars(g)=SUG; Fats(g)=FAT; Sodium(mg)=NA
Screening 1: Regulation 1169/2011=R1; Royal Decree 1487/2009=R2; Regulation 432/2012=R3; World Anti-Doping Code=R5
Screening 2: Amount of protein recommended dose=AP; Limiting amino acid=LA
Screening 3: Others manufacture's intake recommendation=OR; Allergens=AG; Enzymes=EZY; Additives=ADD; B12 (μg)=B12
Use=USE; Serving=SG; Degree of evidence(Australian Institute of Sport)=DE; Certification=CT; Frequency=FC; Source of information=SI
✓ Complies with what is established in the legislation.

PR	CH	KCAL	PROT	AA	BCAA	HC	SUG	FAT	NA
Vegan vanilla flavor protein (pea and rice protein isolate)	Weider	427	77	No claims	No claims	5,6	0,23 g	8,5	1840

Screening 1					Screening 2		Screening 3				
R1	R2	R3	R4	R5	AP	LA	OR	AG	EZY	ADD	B12
✓	✓	"Development and care of muscle mass" ✓	✓	No claims	23,1	No claims	No claims	Oats and wheat	No claims	Oatmeal<br>Quinoa<br>Buckwheat<br>Amaranth<br>Buckwheat<br>Sprouted wheat<br>Millet<br>Alfalfa<br>Chlorella Spirulina	2,75

SG	DE	CT	FC	SI
30 g	A	No		Label

Abbreviations: Product(100gr)=PR; Comercial House=CH; Kilocalories=Kcal; Protein(g)=PROT; Aminogram(g)=AA; BCAA(g)=BCAA; Carbohydrates(g)=HC; Of which sugars(g)=SUG; Fats(g)=FAT; Sodium(mg)=NA
Screening 1: Regulation 1169/2011=R1; Royal Decree 1487/2009=R2; Regulation 432/2012=R3; World Anti-Doping Code=R5
Screening 2: Amount of protein recommended dose=AP; Limiting amino acid=LA
Screening 3: Others manufacture's intake recommendation=OR; Allergens=AG; Enzymes=EZY; Additives=ADD; B12 (µg)=B12
Use=USE; Serving=SG; Degree of evidence(Australian Institute of Sport)=DE; Certification=CT; Frequency=FC; Source of information=SI
✓ Complies with what is established in the legislation.

PR	CH	KCAL	PROT	AA	BCAA	HC	SUG	FAT	NA
Vegan protein flavor mix of berries (pea and rice protein isolate)	Weider	426	75	No claims	No claims	7	0,7 g	8,5	1716

SG	DE	CT	FC	SI
30 g	A	No	No claims	Label

Screening 1					Screening 2		Screening 3				
R1	R2	R3	R4	R5	AP	LA	OR	AG	EZY	ADD	B12
✓	✓	No claims	No claims	No claims	22,5	No claims	No claims	Oats and wheat	No claims	Oatmeal Quinoa Buckwheat Amaranth Buckwheat Sprouted wheat Millet Alfalfa Chlorella Spirulina Maltodextrin Aromas Stevia Dyes (turmeric, pepper extracts, lime, spinach, broccoli, ginger, kale, cabbage, green tea, raspberry, banana, kiwi, mango, plum, blueberry, pineapple, orange, grapefruit, cauliflower, lemon, cherry) Gum arabic	2,75

Abbreviations: Product(100gr)=PR; Comercial House=CH; Kilocalories=Kcal; Protein(g)=PROT; Aminogram(g)=AA; BCAA(g)=BCAA; Carbohydrates(g)=HC; Of which sugars(g)=SUG; Fats(g)=FAT; Sodium(mg)=NA
Screening 1: Regulation 1169/2011=R1; Royal Decree 1487/2009=R2; Regulation 432/2012=R3; World Anti-Doping Code=R5
Screening 2: Amount of protein recommended dose=AP; Limiting amino acid=LA
Screening 3: Others manufacture's intake recommendation=OR; Allergens=AG; Enzymes=EZY; Additives=ADD; B12 (μg)=B12
Use=USE; Serving=SG; Degree of evidence(Australian Institute of Sport)=DE; Certification=CT; Frequency=FC; Source of information=SI
✓ Complies with what is established in the legislation.

PR	CH	KCAL	PROT	AA	BCAA	HC	SUG	FAT	NA
Vegetable protein (pea, rice, pumpkin and hemp protein powder)	El Granero Integral	416,6	77	No claims	No claims	1,8	1,14 g	8,7	520

SG	DE	CT	FC	SI
20 g	A	No		Label

Screening 1					Screening 2		Screening 3				
R1	R2	R3	R4	R5	AP	LA	OR	AG	EZY	ADD	B12
✓	✓	No claims	No claims	No claims	15,4	No claims	No claims	May contain traces of gluten, soy, sesame and nuts	No claims	No claims	No claims

Abbreviations: Product(100gr)=PR; Comercial House=CH; Kilocalories=Kcal; Protein(g)=PROT; Aminogram(g)=AA; BCAA(g)=BCAA; Carbohydrates(g)=HC; Of which sugars(g)=SUG; Fats(g)=FAT; Sodium(mg)=NA
Screening 1: Regulation 1169/2011=R1; Royal Decree 1487/2009=R2; Regulation 432/2012=R3; World Anti-Doping Code=R5
Screening 2: Amount of protein recommended dose=AP; Limiting amino acid=LA
Screening 3: Others manufacture's intake recommendation=OR; Allergens=AG; Enzymes=EZY; Additives=ADD; B12 (µg)=B12
Use=USE; Serving=SG; Degree of evidence(Australian Institute of Sport)=DE; Certification=CT; Frequency=FC; Source of information=SI
✓ Complies with what is established in the legislation.

PR	CH	KCAL	PROT	AA	BCAA	HC	SUG	FAT	NA
Hemp protein powder	El Granero Integral	358	50	No claims	No claims	5	4,1 g	11	No claims

SG	DE	CT	FC	SI
20 g	A	No	No claims	Label

Screening 1					Screening 2		Screening 3				
R1	R2	R3	R4	R5	AP	LA	OR	AG	EZY	ADD	B12
✓	✓	No claims	No claims	No claims	10	No claims	No claims	May contain traces of gluten, soy, sesame and nuts	No claims	No claims	No claims

Abbreviations: Product(100gr)=PR; Comercial House=CH; Kilocalories=Kcal; Protein(g)=PROT; Aminogram(g)=AA; BCAA(g)=BCAA; Carbohydrates(g)=HC; Of which sugars(g)=SUG; Fats(g)=FAT; Sodium(mg)=NA
Screening 1: Regulation 1169/2011=R1; Royal Decree 1487/2009=R2; Regulation 432/2012=R3; World Anti-Doping Code=R5
Screening 2: Amount of protein recommended dose=AP; Limiting amino acid=LA
Screening 3: Others manufacture's intake recommendation=OR; Allergens=AG; Enzymes=EZY; Additives=ADD; B12 (μg)=B12
Use=USE; Serving=SG; Degree of evidence(Australian Institute of Sport)=DE; Certification=CT; Frequency=FC; Source of information=SI
✓Complies with what is established in the legislation.

PR	CH	KCAL	PROT	AA	BCAA	HC	SUG	FAT	NA
Vegan vegetable protein chocolate flavor (pea protein isolate)	Hero tech nutrition	325,33	79,18	Alanine 4,04 Arginine 6,68 Aspartic acid 9,55 Cysteine 0.62 Glutamic acid 10,48 Glycine 3,93 Histidine 1,86 Isoleucine 4,35 Leucine 6.29 Lysine 5.05 Methionine 1,16 Phenylalanine 4,78 Proline 3,61 Serine 3.34 Threonine 1,24 Tyrosine 2.48 Valine 4,04	13,68	0,69	0,04 g	0,65	348

SG	DE	CT	FC	SI
30 g	A	No	No claims	Label

Screening 1					Screening 2		Screening 3				
R1	R2	R3	R4	R5	AP	LA	OR	AG	EZY	ADD	B12
✓	✓	"It contributes to the growth and maintenance of muscle mass and also to the maintenance of bones in normal conditions" ✓	✓	No claims	23,754	Tryptophan	No claims	May contain traces of egg, soy, sulfur dioxide, sulphites, milk, mollusks, crustaceans and fish	No claims	Cocoa powder Aromas Sodium chloride Acesulfame K Sucralose	No claims

Abbreviations: Product(100gr)=PR; Comercial House=CH; Kilocalories=Kcal; Protein(g)=PROT; Aminogram(g)=AA; BCAA(g)=BCAA; Carbohydrates(g)=HC; Of which sugars(g)=SUG; Fats(g)=FAT; Sodium(mg)=NA
Screening 1: Regulation 1169/2011=R1; Royal Decree 1487/2009=R2; Regulation 432/2012=R3; World Anti-Doping Code=R5
Screening 2: Amount of protein recommended dose=AP; Limiting amino acid=LA
Screening 3: Others manufacture's intake recommendation=OR; Allergens=AG; Enzymes=EZY; Additives=ADD; B12 (μg)=B12
Use=USE; Serving=SG; Degree of evidence(Australian Institute of Sport)=DE; Certification=CT; Frequency=FC; Source of information=SI
✓Complies with what is established in the legislation.

PR	CH	KCAL	PROT	AA	BCAA	HC	SUG	FAT	NA
Pea protein, chocolate flavored rice protein	Tegor sport nutrition	No claims	76	Glutamic acid 12,7<br>Aspartic acid 8,45<br>Proline 6,4<br>Leucine 6,13<br>Arginine 5,88<br>Lysine 5,32<br>Alanine 4,3<br>Valine 3.88<br>Phenylalanine 3.86<br>Tyrosine 3.8<br>Threonine 3.8<br>Isoleucine 3.33<br>Glycine 3,02<br>Serine 3<br>Histidine 2,5<br>Methionine 1,1<br>Cysteine 1<br>Tryptophan 1	13,34	3,4	No claims	5,2	No claims

SG	DE	CT	FC	SI
20-60 g (about 40)	A	No	No claims	Label

Screening 1					Screening 2		Screening 3				
R1	R2	R3	R4	R5	AP	LA	OR	AG	EZY	ADD	B12
✓	✓	"Development of muscle mass" ✓	✓	No claims	30,4	No claims	For athletes in the rest phase	No claims	No claims	Mint aroma<br>Cocoa<br>Moringa dry extract<br>Quinoa	No claims

Abbreviations: Product(100gr)=PR; Comercial House=CH; Kilocalories=Kcal; Protein(g)=PROT; Aminogram(g)=AA; BCAA(g)=BCAA; Carbohydrates(g)=HC; Of which sugars(g)=SUG; Fats(g)=FAT; Sodium(mg)=NA
Screening 1: Regulation 1169/2011=R1; Royal Decree 1487/2009=R2; Regulation 432/2012=R3; World Anti-Doping Code=R5
Screening 2: Amount of protein recommended dose=AP; Limiting amino acid=LA
Screening 3: Others manufacture's intake recommendation=OR; Allergens=AG; Enzymes=EZY; Additives=ADD; B12 (µg)=B12
Use=USE; Serving=SG; Degree of evidence(Australian Institute of Sport)=DE; Certification=CT; Frequency=FC; Source of information=SI
✓ Complies with what is established in the legislation.

PR	CH	KCAL	PROT	AA	BCAA	HC	SUG	FAT	NA
Pea protein, chocolate flavored rice protein	Pro cell	396,47	72,05	No claims	No claims	13,74	0,5 g	5,67	88

SG	DE	CT	FC	SI
50 g	A	No	No claims	Label

Screening 1					Screening 2		Screening 3				
R1	R2	R3	R4	R5	AP	LA	OR	AG	EZY	ADD	B12
✓	✓	No claims	No claims	No claims	36,025	No claims	No claims	No claims	No claims	Maltodextrin<br>BCAA's 2:1:1 (leucine, isoleucine, valine)<br>Aromas<br>Natural cocoa<br>Sodium chloride<br>Sucralose splenda<br>Vitamin B6<br>Vitamin B12	7,5

Abbreviations: Product(100gr)=PR; Comercial House=CH; Kilocalories=Kcal; Protein(g)=PROT; Aminogram(g)=AA; BCAA(g)=BCAA; Carbohydrates(g)=HC; Of which sugars(g)=SUG; Fats(g)=FAT; Sodium(mg)=NA
Screening 1: Regulation 1169/2011=R1; Royal Decree 1487/2009=R2; Regulation 432/2012=R3; World Anti-Doping Code=R5
Screening 2: Amount of protein recommended dose=AP; Limiting amino acid=LA
Screening 3: Others manufacture's intake recommendation=OR; Allergens=AG; Enzymes=EZY; Additives=ADD; B12 (µg)=B12
Use=USE; Serving=SG; Degree of evidence(Australian Institute of Sport)=DE; Certification=CT; Frequency=FC; Source of information=SI
✓ Complies with what is established in the legislation.

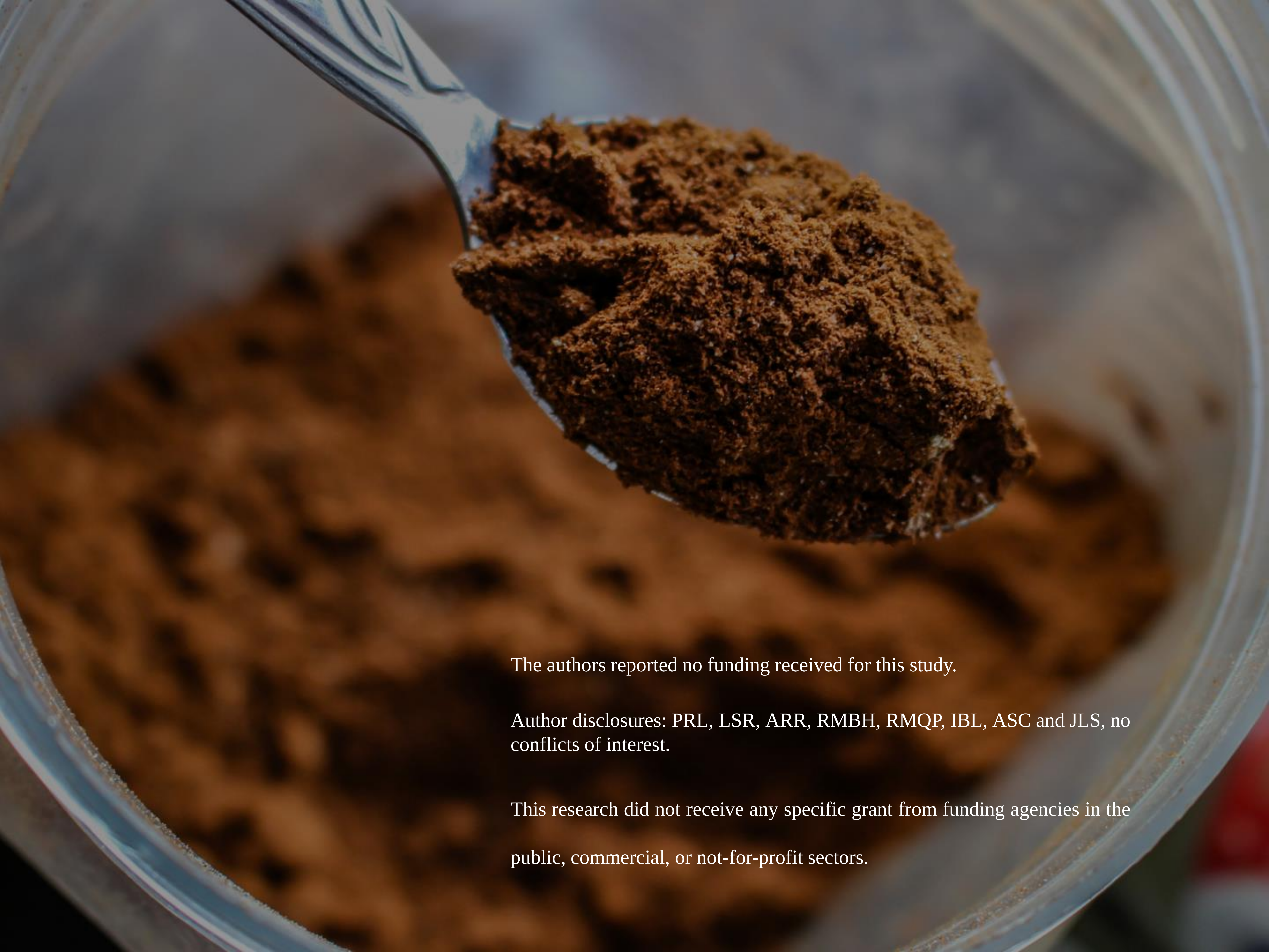

The authors reported no funding received for this study.

Author disclosures: PRL, LSR, ARR, RMBH, RMQP, IBL, ASC and JLS, no conflicts of interest.

This research did not receive any specific grant from funding agencies in the public, commercial, or not-for-profit sectors.
